# Characterisation of the thermophilic P450 CYP116B305 identified using metagenomics-derived sequence data from an Australian hot spring

**DOI:** 10.1007/s00253-025-13521-2

**Published:** 2025-05-31

**Authors:** Simran Kundral, Peter D. Giang, Leah R. Grundon, Jenna M. Supper, Sunil K. Khare, Paul V. Bernhardt, Paul Evans, Stephen G. Bell, James J. De Voss

**Affiliations:** 1 The University of Queensland, Australia - Indian Institute of Technology Delhi Research Academy (UQIDRA), Delhi, India; 2 School of Chemistry and Molecular Biosciences, The University of Queensland, Brisbane, 4072 Australia; 3 Enzyme and Microbial Biochemistry Laboratory, Department of Chemistry, Indian Institute of Technology Delhi, New Delhi, India; 4 Australian Centre for Ecogenomics, School of Chemistry and Molecular Biosciences, University of Queensland, Brisbane, 4072 Australia; 5 Department of Biological Sciences, Indian Institute of Science Education and Research Kolkata, Kolkata, India; 6 Department of Chemistry, The University of Adelaide, Adelaide, SA 5005 Australia

**Keywords:** CYP116B305, Cytochrome P450, Innot Hot Springs

## Abstract

**Abstract:**

Cytochrome P450 enzymes (P450s) have gained significant attention due to their remarkable ability to oxidise unactivated C-H bonds with high regio- and stereoselectivity. Their industrial utility is often limited by challenges such as low stability, poor expression, and dependence on elusive redox partners. These issues have driven the search for more robust P450s, especially those that are inherently stable under extreme conditions typical of industrial processes. “Self-sufficient P450s” in which the P450 heme domain is naturally fused to redox domains in a single polypeptide chain eliminates the need to identify and separately express required redox partners. Furthermore, P450s from thermophilic organisms are more temperature tolerant with fewer stability issues. This study presents a self-sufficient P450, CYP116B305, identified from metagenomically assembled genomes from Innot Hot Springs (71 °C), located in North Queensland, Australia. *CYP116B305* was heterologously expressed in *Escherichia coli* and purified using standard protocols. Investigation of the thermal stability of CYP116B305 revealed a robust heme domain with a ^15^*T*_50_ value of 56.9 ± 0.1 °C, while the reductase domain exhibited slightly lower stability, with a ^15^*T*_50_ value of 52.5 ± 0.5 °C. Further characterisation revealed that CYP116B305 efficiently bound to and hydroxylated 2-hydroxyphenylacetic acid (2-HPA) at the C-5 position, yielding homogentisic acid. The catalytic parameters, including the coupling efficiency and rate of electron transfer from the NADPH cofactor to the P450 heme, were shown to improve at an elevated temperature (45 °C) compared to 25 °C. The combination of the self-sufficiency and improved stability makes CYP116B305 a promising platform for biotechnological applications and biocatalyst engineering.

**Key points:**

• *Hot spring metagenomics reveals thermostable P450s of biocatalytic value*.

• *CYP116B305 shows enhanced catalytic activity at elevated temperature (45 °C)*.

• *CYP116B305 is a promising platform enzyme for diverse biotechnological use*.

**Graphical Abstract:**

**Figure:**
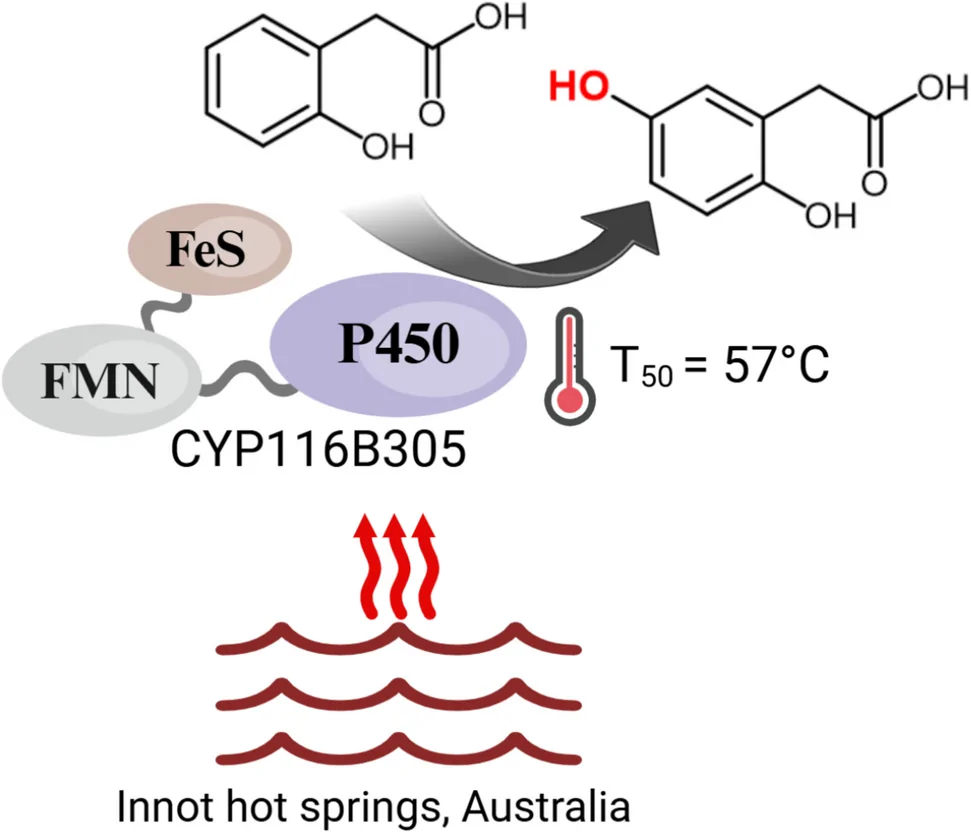

**Supplementary Information:**

The online version contains supplementary material available at 10.1007/s00253-025-13521-2.

## Introduction

Cytochromes P450 enzymes (CYPs; P450s) constitute a vast and diverse superfamily of heme-containing monooxygenases found in all kingdoms of life. The remarkable ability of CYP enzymes to perform synthetically challenging oxidation of inert C-H bonds under mild conditions with high regio-, stereo-, and enantioselectivity makes them highly desirable biocatalysts (Roiban and Reetz 2015; Urlacher and Girhard 2019).

**Fig. 1:**
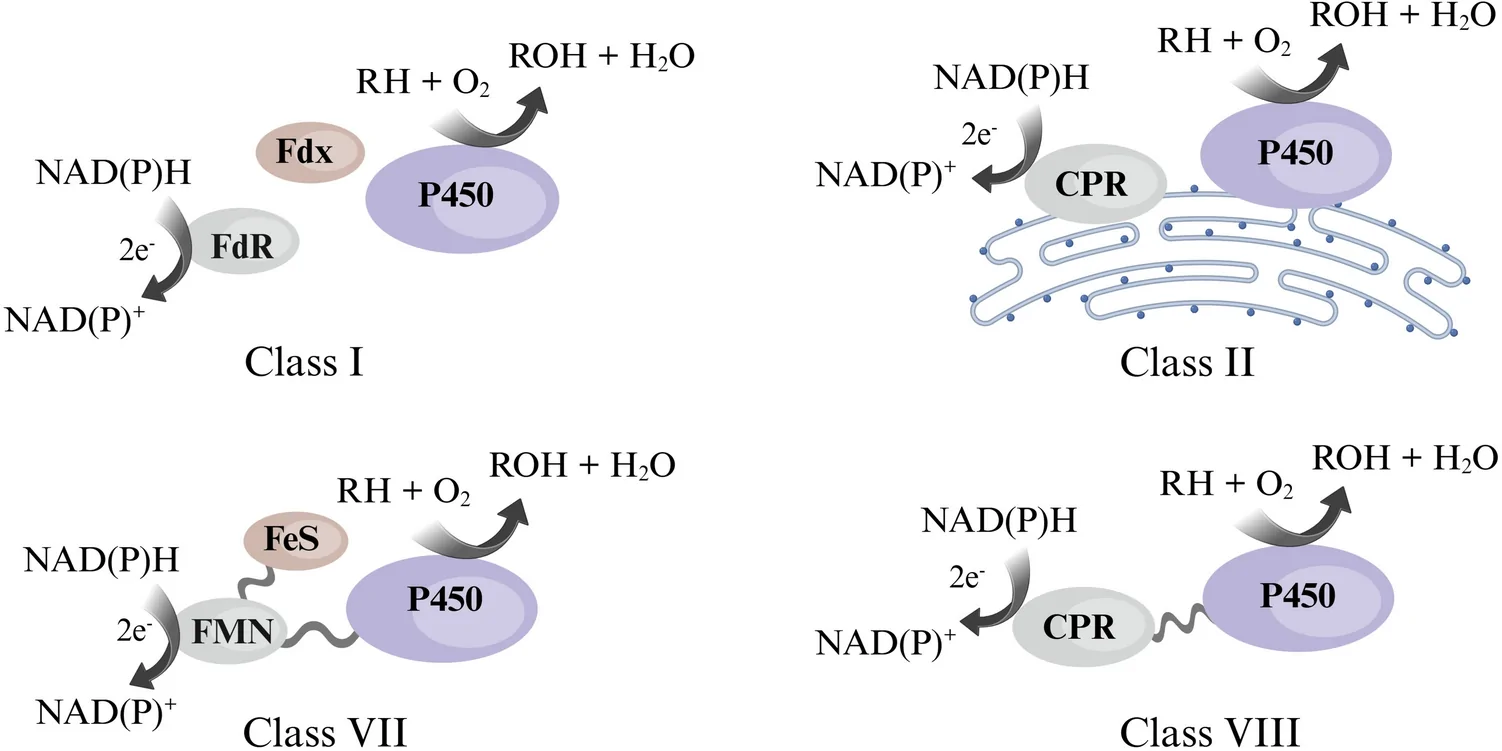
Class-type classification of CYPs based on the type and arrangement of redox partner proteins. Shown here are common classes I, II, VII, and VIII. However, CYPs are categorised into a total of ten classes (Finnigan et al. 2020)

Typically, the monooxygenase activity of CYPs depends on the activation and reductive scission of dioxygen bound to the heme iron. This requires association with one or more auxiliary redox partners to shuttle the electrons derived from NAD(P)H. Based on the nature of these redox partners, CYPs have been categorised into classes (Fig. 1), with classes I and II being the most common. There has been more diversity discovered in redox partners supporting specific CYPs, and this has led to a broader classification system with ten classes (Hannemann et al. 2007; Finnigan et al. 2020).

Electron transfer through redox partners is often considered the rate-limiting step in CYP-driven catalysis, attributed to their redox potentials and orientation of and distance between the redox partners and the heme domain (Poulos and Follmer 2022). Bacteria often contain multiple ferredoxin and ferredoxin reductase genes that are usually not located near the CYP gene, making it difficult to identify native redox partners. The reconstitution of bacterial CYPs with surrogate redox partners is a long-standing practise; however, it often leads to reactions with low catalytic efficiency (Momoi et al. 2006; Giang et al. 2024). However, this limitation can be overcome by using catalytically self-sufficient CYPs, wherein the heme and redox domains are naturally fused into a single polypeptide chain. This fusion arrangement is observed in class VII and class VIII P450 s (Fig. 1), and these CYPs often exhibit higher electron transfer rates and better coupling efficiency than their multi-component counterparts (Eser et al. 2021). For instance, CYP102 A1 (P450_BM3_) from *Bacillus megaterium* exhibits the highest P450 activity reported, i.e. 17,000 min^−1^ (for arachidonate hydroxylation) (Davis et al. 1996; Noble et al. 1999).

In addition to the CYP102 family, the CYP116 family (in class VII) also constitutes self-sufficient enzymes with naturally fused heme and redox domains. CYP116 family enzymes contain a [2 Fe-2S] cluster containing ferredoxin and an FMN-containing flavin domain, which altogether constitutes a so-called phthalate family oxygenase reductase (PFOR) domain fused to the catalytic heme domain (Roberts et al. 2002). From this family, CYP116B2 (P450 RhF) is one of the first discovered and most well-characterised self-sufficient enzymes. It was isolated from *Rhodococcus* sp. NCIMB172 (Roberts et al. 2003) and is reported to catalyse a range of oxidative reactions, such as O- and N-dealkylations, aromatic hydroxylation, and asymmetric sulfoxidations (O’Reilly et al. 2013). Several other homologs of this enzyme have also been identified and characterised (Warman et al. 2012; Yin et al. 2014; Minerdi et al. 2015; Tavanti et al. 2018b; Porter et al. 2018). Though the identification of new CYP116B enzymes has dramatically increased, little information is available on their natural substrates. Recently, another homolog of P450RhF, OhpA from *Cupriavidus pinatubonensis*, was suggested to be involved in the catabolism of 2-hydroxyphenylacetic acid (2-HPA) via the homogentisate pathway, providing evidence for the natural function of a CYP116B member (Donoso et al. 2021). We have also recently reported another homolog, CYP116B234, from *Rhodococcus globerulus* that naturally catalyses 2-HPA hydroxylation, as suggested by proteomic analysis (Kundral et al. 2025). Interestingly, CYP116B46 from *Tepidiphilus thermophilus* has also been shown to efficiently oxidise 2-HPA to homogentisic acid (Akter et al. 2024).

One of the major challenges in the commercial deployment of CYP enzymes is their intrinsic fragility at higher temperatures, typical of industrial processes, which limits their application as biocatalysts for large-scale production. Therefore, CYPs derived from thermophiles have been given attention owing to their likely robustness and stability, which are required for commercial applications. Moreover, improved stability also extends biocatalyst lifetime, facilitates long-term storage, and reduces transportation costs, all of which are critical for cost-effective biocatalytic processes. To date, a limited number of thermostable CYPs have been studied and characterised, including five from the CYP116 family, namely CYP116B46, CYP116B29, CYP116B63, CYP116B64, and CYP116B65 with *T*_m_ values ranging from 45 to 60 °C (Tavanti et al. 2018b).

Integrating self-sufficiency with improved thermal stability makes the CYP116 family a source of promising biocatalysts. However, studies have indicated that the heme and reductase domains of self-sufficient enzymes might have evolved differently in terms of their thermal stability (Munro et al. 1996; Jamakhandi et al. 2005; Tavanti et al. 2018b). Therefore, it is important to evaluate the thermal stability of both domains to ensure effective electron transfer from the reductase to the heme domain in self-sufficient CYPs at higher temperatures.

For more comprehensive evaluations of stability to occur, heat stable enzymes need to be identified from these extreme environments. However, this is difficult as the majority of microorganisms from these environments remain uncultivated. Therefore, the use of bioinformatic tools to discover novel enzymes from microbially derived DNA sequence data has become a favoured approach (Achudhan et al. 2024; Hogg et al. 2024). These analysis techniques also allow for the identification of rare sequence variants linked to microbial taxonomy through assembly of genomes from the metagenomic data. In this work, DNA sequence data was generated from microbial biomass collected at the Innot Hot Springs (North Queensland, Australia) from locations with surface temperatures between 68 and 71 °C and neutral to alkaline pH (7.2–8.1). Sequence data generated from these thermal springs was subjected to assembly, binning, and mining using “bioprospecting” tools to reveal a self-sufficient P450 monooxygenase (CYP116B305) present in the metagenomic database of the Innot Hot Springs. CYP116B305 was identified from metagenome-assembled genomes (MAGs) associated with unnamed genera of the Geminicoccaceae family, a member of the Pseudomonadota phylum and class Alphaproteobacteria. To date, CYP116B305 represents the first reported member of the CYP116 family to be identified from this lineage or its higher taxonomic rank, the order Geminicoccales. Given the critical role of the reductase domain in efficient catalysis by the holoenzyme, we have evaluated the thermal stability of both the heme and reductase domains. We have further investigated the functional role of the CYP116B305 enzyme and its kinetics at elevated temperatures. Our findings contribute to the potential use of metagenome data as a source of new thermostable CYP biocatalysts that can be used as platform enzymes for future engineering studies.

## Experimental section

### Chemicals

Bacto-tryptone was purchased from Thermo Fisher Scientific (Gibco, AU). All the other medium components, chemicals, and solvents used in the study were of analytical grade purity and were purchased from Sigma-Aldrich (Castle Hill, NSW) unless stated otherwise.

### Sample collection and DNA extraction

Approximately 300 mL of 68 °C water was collected at a depth of 100 cm below the gravel surface where the heated water percolates to the surface of the Innot Hot springs (Innot 4872, Queensland, Australia). The collected water was then passed through a 0.22 µm polycarbonate membrane filter, with the filtrate collected for physicochemical measurements such as pH, conductivity, salinity, ion concentrations, and the microbial biomass retained on the 0.22 µm filter frozen on dry ice for later DNA extraction. Subsequent DNA extractions were performed by slicing the frozen polycarbonate filters into small strips and placing them into bead tubes supplied with the Qiagen DNeasy PowerLyzer PowerSoil Kit (#12855) that was then used to extract DNA from filtered microbial biomass.

### Metagenome sequencing, assembly, and annotation

Extracted DNA was subjected to high-throughput sequencing on an Illumina NovaSeq machine. The resulting sequence data were trimmed using Trimmomatic (Bolger et al. 2014), assembled using metaSPAdes (Nurk et al. 2017) and binned with MetaBAT2 (Kang et al. 2019) within the Aviary software suite (Newell et al. 2025) using default settings. Searches for P450 sequences were employed using the GraftM sequence recovery software (Boyd et al. 2018), for which the CYP116B305 was returned from these analyses. Estimation of the community structure using SingleM (Woodcroft et al. 2024) identified the MAG that contained CYP116B305 as being approximately 0.1% total abundance of the microbial community. This MAG, designated SE0867_metabat2_refined_bins.tsv.040, and others from the same dataset have been submitted to the European Nucleotide Archive (ENA) under the project accession number PRJEB88793.

### Identification and phylogenetic analysis

A 2310‐bp ORF, putatively encoding a CYP enzyme, was identified from the Innot Hot Springs metagenomic database. A BLAST search on the UniProt database (The UniProt Consortium et al. 2025) was performed to identify the closest homologs of the identified gene. Amino acid sequence alignment of enzymes from the CYP116B subfamily was conducted using the Clustal Omega programme (Sievers et al. 2011), and a phylogenetic tree was built using the neighbour-joining method within the MEGA11 software (Tamura et al. 2021). The evolutionary distance units, number of amino acid substitutions per site, were calculated using the Poisson correction method.

### Protein expression, purification, and spectroscopic characterisation

The gene encoding the sequence of *CYP116B305* was codon-optimised for *E. coli*, commercially synthesised and cloned into pET-28a(+) plasmid by Twist Biosciences, USA, to form a pET-28a(+)/*CYP116B305* construct. This plasmid was transformed into *E. coli* BL21 (DE3) cells, and the following day, single colonies were used to inoculate Luria–Bertani (LB) medium (4 × 5 mL) containing kanamycin (50 µg mL^−1^) and incubated at 37 °C (200 rpm) overnight. These seed cultures were then used to inoculate Terrific Broth (TB) medium (4 × 500 mL) containing kanamycin (50 µg mL^−1^) and incubated at 37 °C (200 rpm) until OD_600_ reached 0.6–0.8. The incubation temperature was decreased to 25 °C, and 5-aminolevulinic acid (0.5 mM) and riboflavin (50–150 µM) were added to the medium for the protein expression and further incubated for 16 h (120 rpm). Cells were then harvested by centrifugation (5000 × *g*, 20 min at 4 °C) and stored at − 80 °C.

All the purification steps were performed at 4 °C unless otherwise stated. The cell pellet was resuspended in 160 mL of buffer A (100 mM potassium phosphate (KPi), 50 mM NaCl, 5% glycerol, pH 7.4) supplemented with phenyl methyl sulfonyl fluoride (PMSF) (200 µM), DL-dithiothreitol (DTT) (0.5 mM), and lysozyme (0.1 g L^−1^). Cells were lysed by sonication (Branson Sonifier 450) with 3 × 30 s cycles of 1 s on and 1 s off with 30% amplitude, followed by centrifugation (20,000 × *g*, 50 min) to remove the cell debris. The obtained cell lysate was filtered (0.45 µm) and diluted 1/3 with buffer A. Protein purification was performed via affinity chromatography using a Ni–NTA column (5 mL, HisTrap FF, Cytiva) pre-equilibrated with buffer A (5 column volumes (CV)) and connected to an FPLC AKTA pure system (5 mL min^−1^). The cell lysate was loaded on to the Ni–NTA column and washed with buffer A (5 CV). CYP116B305 was eluted with a stepwise gradient of 20–150 mM l-histidine, with P450 containing fractions identified by their red colour, and analysed for purity using UV–visible spectroscopy (Shimadzu UV-1800 spectrophotometer). Fractions with a Reinheitszahl (RZ, absorption ratio *A*_417 nm_/*A*_280 nm_) > 0.70 were combined, concentrated by ultrafiltration (Amicon filtration units) and resuspended in KPi buffer (50 mM, pH 7.4). For storage, protein aliquots were snap-frozen in liquid nitrogen and stored at − 80 °C.

The purified protein was subjected to SDS-PAGE (NuPAGE 4–12%), and the gel was analysed using ImageJ2 software (Image Processing and Analysis in Java) for densitometric analysis, estimating the relative area and integrated density of the protein bands (Schindelin et al. 2015; Rueden et al. 2017). The band of interest was excised from the gel for in-gel tryptic digestion, followed by mass spectrometry analysis as detailed previously (Kundral et al. 2025).

The optical properties of purified protein were measured spectrophotometrically, and P450 concentration was measured by carbon monoxide (CO)–difference spectroscopy following the established protocol of Guengerich et al. (2009), using an extinction coefficient of 91,000 M^−1^ cm^−1^ at 450 nm. In order to quantify the FMN content in the holoenzyme (Chapman and Reid 1999; Kundral et al. 2025), purified protein (20 µM) in KPi buffer (50 mM, pH 7.4) was heat denatured at > 95 °C for 5 min in the dark to release the flavin. The solution was then placed on ice, and the precipitated protein was separated from the yellow-coloured supernatant by centrifugation (13,000 × *g*, 5 min at 4 °C). The concentration of flavin in the supernatant was calculated using an extinction coefficient at 445 nm of 12,500 M^−1^ cm^−1^ (Hawkes et al. 2010).

Measurement of electron transfer from PFOR domain to P450 heme domain was performed by CO-difference spectroscopy as described above. Briefly, a solution containing P450 (1 µM), substrate (2-HPA; 1 mM), and NADPH (2 mM) was prepared in KPi buffer (50 mM, pH 7.4), and a baseline spectrum was recorded. CO gas was then bubbled through the sample, and the UV–visible spectral changes (400–500 nm) were monitored over 10 min. The formation of the CO-bound P450 complex was quantified as described above.

### Determination of thermal stability

The thermal stability of CYP116B305 was determined by measuring the residual intact CYP116B305 by CO-difference spectroscopy after incubation at elevated temperatures compared to the non-incubated protein (retained at 4 °C) (Tavanti et al. 2018b; Harris et al. 2022). To determine the thermal stability of the P450 heme domain, a solution containing purified CYP116B305 (1 µM) in KPi buffer (50 mM, pH 7.4) was incubated at various temperatures (30–70 °C) for 15 and 60 min. The concentration of intact P450 was then measured using CO-difference spectroscopy. The obtained data were fitted to a Hill-type equation (Eq. 1) using GraphPad Prism to extract *T*_50_ values (*T*_m_) for both time points. *T*_50_ refers to the temperature at which 50% of intact P450 remains after the incubation, *y* refers to the residual CO-binding activity at a given temperature *T*, *y*_*0*_ refers to the maximum CO-binding activity of non-incubated protein solution, and *h* is the Hill coefficient.

1 $$\begin{array}{c}y=y_0-\frac{\left({\mathrm y}_0\cdot\mathrm T^{\mathrm h}\right)}{\left(\mathrm T_{50}^{\mathrm h}+\mathrm T^{\mathrm h}\right)}\end{array}$$

The thermostability of the reductase domain in CYP116B305 was evaluated by measuring the residual NADPH-dependent cytochrome *c* reduction activity after incubation at elevated temperatures (30–70 °C) for 15 and 60 min. Enzymatic activity was determined spectrophotometrically by monitoring cytochrome *c* reduction at 550 nm (*ε* = 28,000 M^−1^ cm^−1^) (Guengerich et al. 2009) in KPi buffer (50 mM, pH 7.4) containing CYP116B305 (260 nM), cytochrome *c* (equine heart, Sigma-Aldrich, 40 µM), and NADPH (32 µM).

### Substrate binding analysis

To determine the dissociation constant (*K*_*D*_) for substrates with CYP116B305, difference spectroscopy was performed between a solution of CYP116B305 (2–4 µM) and a solution of the same enzyme with an increasing concentration of the substrate of interest (0–100 µM). The absolute absorbance difference between 418 and 393 nm was plotted against the increasing substrate concentration, and *K*_D_ was calculated using the typical hyperbolic equation (Eq. 2). However, for tight binding substrates, a quadratic equation was applied (Eq. 3), where *A* is the absorbance difference between 393 and 418 nm, *A*_max_ is the maximal absorbance difference between 393 and 418 nm, [*E*_*T*_] is the concentration of CYP116B305, and [*S*_*T*_] is the total substrate concentration.

2 $$\begin{array}{c}V=\frac{\Delta V\mathrm{max}\times\left[S\right]}{K_D+\left[S\right]}\end{array}$$

The heme iron spin-state shift was estimated by comparison with a set of spectra generated from the sum of the appropriate percentages of the spectra of the substrate-free form (> 95% low spin, Soret peak at 422 nm) and the (*S*)-limonene bound form (100% high spin, Soret peak at 390 nm) of CYP108 N12 (Giang et al. 2022).

3 $$\begin{array}{c}\Delta A=\Delta A_{max}\frac{\left(\left[E_T\right]+\left[S_T\right]+K_D\right)-\sqrt{\left(\left[E_T\right]+\left[S_T\right]+K_D\right)^2 - 4\left[E_T\right]\left[S_T\right]}}{2\left[E_T\right]}\end{array}$$

### In silico modelling and docking studies

The full-length structure of CYP116B305 was predicted using AlphaFold 3 programme (Abramson et al. 2024) and visualised in PyMOL (Schrödinger, LLC 2021). Substrate docking was performed using AutoDock Vina (Eberhardt et al. 2021), and protein–ligand interactions were predicted using PLIP (Salentin et al. 2015).

### Cofactor preference and kinetics

The cofactor preference of CYP116B305 was determined using the standard procedure of NAD(P)H consumption assays (Meharenna et al. 2008). Briefly, the reaction mixtures contained CYP116B305 (1 µM), catalase (1 µM), and substrate (250 µM) in KPi buffer (50 mM, pH 7.4). The baseline at 340 nm was recorded, the reactions were initiated by the addition of NADPH (0.5–200 µM) or NADH (40–200 µM), and the decrease of absorbance at 340 nm (*ε* = 6220 M^−1^ cm^−1^) was recorded over 1 min at 25 °C. The consumption rates were calculated using nonlinear regression (GraphPad Prism), fitting to either the Michaelis–Menten or a quadratic velocity equation to determine the apparent *K*_M_ and *k*_cat_ values. To assess the effect of temperature, the highest observed NAD(P)H oxidation rates were also measured at 45 °C using NAD(P)H (200 µM) under identical reaction conditions.

Similarly, a cytochrome *c* reduction assay was also performed to derive the kinetic parameters, following established protocols (Guengerich et al. 2009; Tavanti et al. 2018b). Reactions contained cytochrome *c* (equine heart, Sigma-Aldrich, 50 µM), CYP116B305 (200 nM), catalase (1 µM), and varying concentrations of NADPH (0.5–200 µM). Cytochrome *c* reduction was monitored as described above, and nonlinear regression analysis (GraphPad Prism) was utilised to determine the *K*_M_ and *k*_cat_ values.

### In vitro catalytic turnovers

The reaction mixtures contained CYP116B305 (1 µM) in KPi buffer (50 mM, pH 7.4), catalase (1 µM), substrate (600 µM), and NAD(P)H (1.2 mM). Reactions were conducted at both room temperature (25 °C) and elevated temperature (45 °C) under constant stirring for 1 h. A control reaction, omitting the P450 enzyme, was also performed. Post-incubation, reaction mixtures were acidified to pH 0–2 with HCl (except for 4-methoxyacetophenone assays), followed by extraction with ethyl acetate (3 × 1 mL). The combined organic phases were dried over magnesium sulphate and concentrated to 100 µL under a stream of nitrogen gas. Concentrated samples were derivatised with *N*,*O*-bis(trimethylsilyl)trifluoroacetamide (BSTFA-TMS) before being analysed by gas chromatography–mass spectrometry (GC–MS; Zebron ZB-5MS column 30 m × 0.25 mm × 0.25 µm). The method was 50 °C for 5 min followed by increments of 16 °C min^−1^ until 250 °C was reached, then held for 5 min.

For coupling efficiency analysis, the NAD(P)H concentration was reduced to 200 µM, and an internal standard (100 µM) was added to the reactions prior to acidification and extraction. Product quantification (substrate consumption) for 2-HPA and 3-(2-hydroxyphenyl)propionic acid (HPPA) was performed using a standard curve generated from substrate concentrations (0.05–1 mM) in the presence of an internal standard (100 µM). In the 2-HPA reaction, HPPA was used as the internal standard, while in the HPPA reaction, 2-HPA served as the internal standard. In the case of 4-methoxyacetophenone, the reactions were stirred for 2 h, and product formation (4-hydroxyacetophenone) was then quantified using a standard curve generated from 4-hydroxyacetophenone (0.05–1 mM) and an internal standard (fluorene; 100 µM).

### Redox characterisation

A spectroelectrochemical quartz cell (Pine Instruments, 1.7 mm path length) containing a gold “honeycomb” working and counter electrode and a gold with separate Ag/AgCl reference electrode calibrated with quinhydrone (E’ + 288 mV vs normal hydrogen electrode (NHE) at pH 7) was utilised. The reaction cell contained approximately 600 µL of solution (50 mM KPi, pH 7.4), with substrate-free (66 µM CYP116B305) or substrate-bound (17 µM CYP116B305 and 100 µM of 2-HPA). The following mediator complexes (36 µM) were used: [Fe(*trans*-diammac)]^3+^, [Co(AMMEN_4_S_2_sar)]^3+^, [Co(AMMEN_5_Ssar)]^3+^, [Co(sep)]^3+^, [Co(AMMEsar)]^3+^], and [Co(ClMeClabsar)]^3+^ (Scheme S1). These mediator complexes provide redox buffering across − 35 to − 450 mV vs NHE and have small extinction coefficients (*ε* < 300 M^−1^ cm^−1^), which avoids any significant contribution to the UV–visible absorption spectra observed (Bernhardt et al. 2006).

Potential dependent UV–visible absorption spectra were measured over 300–800 nm (Agilent 8453 diode array UV–visible spectrophotometer with temperature controlled) by a Huber Ministat 230 Cooling Bath Circulation Thermostat. Potentials were set and controlled by a potentiostat (Gamry Interface 1010) in the constant potential coulometry mode.

Absorption spectra were recorded when absorbance changes ceased (2–10 min). Spectra were measured at 25 mV intervals with decreasing potential followed by interspaced 25 mV intervals with increasing potential, resulting in 12.5 mV intervals with reversibility. All wavelengths of the potential-dependent absorbance spectra were analysed and modelled with Reactlab Redox (M. Maeder, P. King 2016) using a single (Eq. 4) or double electron transfer model dependent on the cofactor being measured.

For two electron redox reactions, Eq. 5 applies where *E*1 and *E*2 are the potentials of the first and second reductions. *A*_ox_, *A*_int_, and *A*_red_ are the absorbances at a given wavelength of the three redox states.

4 $$\begin{array}{c}Absorbance=\frac{\left({\mathrm A}_{\mathrm{ox}}10^\frac{\mathrm n\left(\mathrm E-\mathrm E^0\right)}{59}+{\mathrm A}_{\mathrm{red}}\right)}{1+10^\frac{\mathrm n\left(\mathrm E-\mathrm E^0\right)}{59}}\end{array}$$

5 $$\begin{array}{c}Absorbance=\frac{\left({\mathrm A}_{\mathrm{ox}}10^\frac{\left(\mathrm E-\mathrm E1\right)}{0.059}+{\mathrm A}_{\mathrm{int}}+{\mathrm A}_{\mathrm{red}}10^\frac{\left(\mathrm E2-\mathrm E\right)}{0.059}\right)}{1+10^\frac{\left(\mathrm E-\mathrm E1\right)}{0.059}+10^\frac{\left(\mathrm E2-\mathrm E\right)}{0.059}}\end{array}$$

## Results

### Discovery of self-sufficient CYP116B305 from Geminicoccaceae

**Fig. 2:**
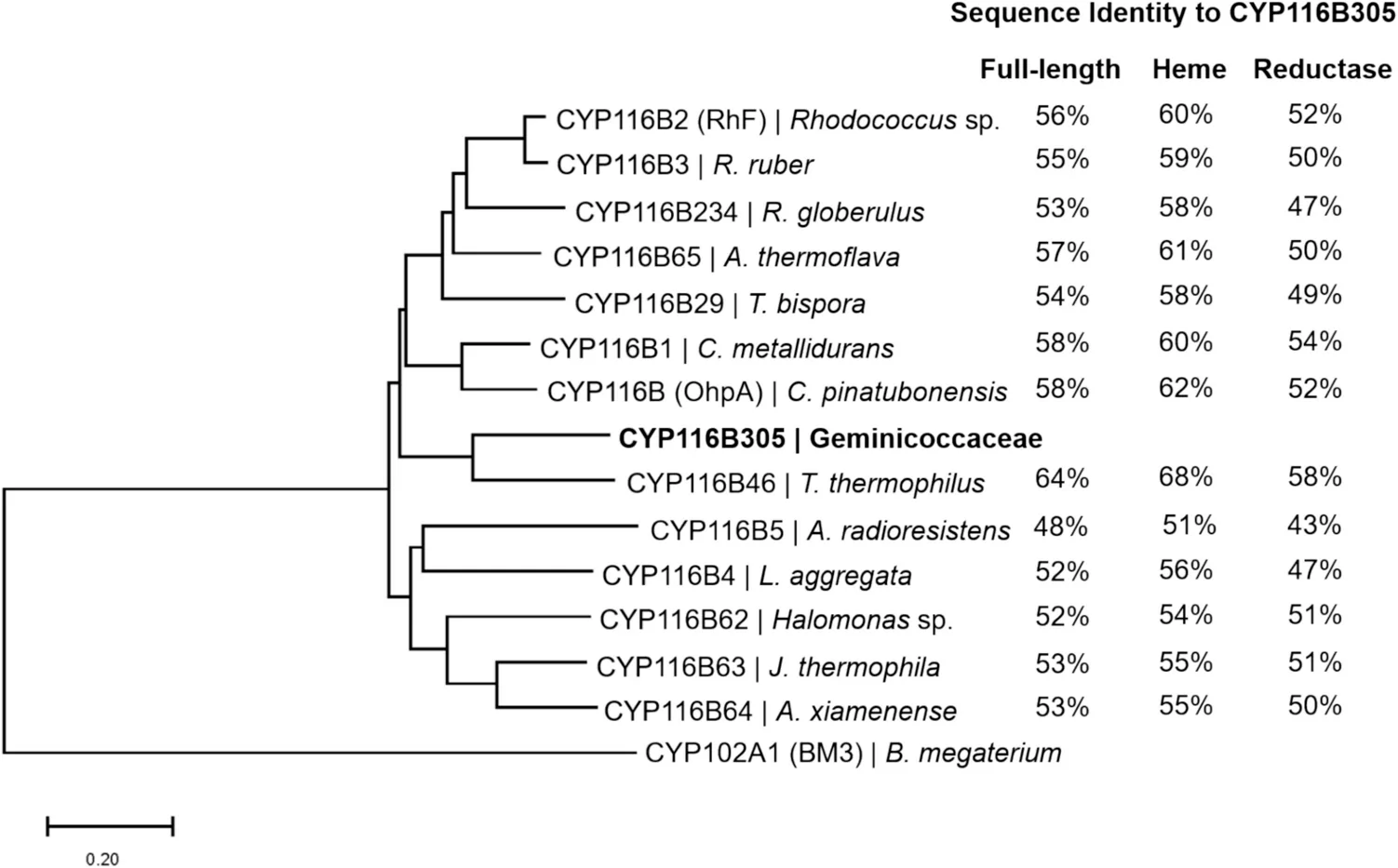
Phylogenetic analysis of the amino acid sequence of CYP116B305 with selected class VII self-sufficient P450 s. Sequence alignment was performed by using Clustal Omega, with evolutionary history inferred by the neighbour-joining method and visualised in MEGA11

From the metagenomic data, a single, putative self-sufficient cytochrome P450 encoding gene was identified in a genome classified as belonging to the family Geminicoccaceae. The protein sequence alignment analysis revealed that the identified gene of 2310 bp exhibited a significant protein sequence identity (> 55%) to CYP116B subfamily members, firmly establishing it as a new member of this subfamily, and it was subsequently classified as CYP116B305 (Fig. S1) (Nelson 2009). The heme domain of CYP116B305 shared the highest protein sequence identity (68%) to the known thermostable CYP116B46 from *T. thermophilus* (Fig. 2) (Tavanti et al. 2018b). The reductase and ferredoxin region of CYP116B305 also superimpose well with the PFOR domain (FMN-Fe-S) of CYP116B46 (58% identity), suggesting a similar domain arrangement and electron transfer pathway operates in CYP116B305.

Studies on thermophilic proteins have identified key compositional trends that can be linked to thermal adaptation, such as lower Ala content, higher Ile content, and a higher ratio of charged residues to polar uncharged residues compared to mesophilic proteins (Harris et al. 2018). While some of these compositional characteristics suggest that CYP116B305 may possess enhanced thermal stability (Table S1), other complex structural characteristics could further contribute to its stability (see the “Supplementary information” section). Therefore, experimental validation was necessary to confirm its thermostability.

### Heterologous expression, purification, and spectroscopic characterisation of CYP116B305

Multiple expression conditions were tested to optimise heterologous CYP116B305 production in *E. coli*. Variations included changing IPTG concentration (0–1 mM), 5-ALA concentration (0.1–0.5 mM), and post-induction temperature (18–30 °C). As analysed by CO-difference spectroscopy, CYP116B305 expression was highest (approximately 200 nmol L^−1^ of culture) in the absence of IPTG but in the presence of 5-ALA (0.5 mM) and riboflavin (50 µM, precursor of coenzyme FMN) supplemented post-induction at 25 °C. Notably, the addition of IPTG, at as little as 50 µM, decreased the expression level by 45%, indicating that the “leaky” expression of the gene in the absence of IPTG provided the highest yield (Mierendorf et al. 1997; Rosano and Ceccarelli 2014; Correddu et al. 2023). The overexpressed CYP116B305 was purified using affinity chromatography. UV–visible spectroscopy was used to determine the RZ (*A*_418_/*A*_280_) a typical purity measure for P450 s, yielding a value of 0.82 (Fig. S2). Protein purity was further assessed via SDS-PAGE analysis, which revealed two bands: one at the expected mass of 85 kDa and another at approximately 53 kDa (Fig. S3). Densitometric analysis showed relative band intensities of ~ 56% and ~ 43%, respectively. Mass spectrometric analysis, following in-gel tryptic digestion, identified the latter band (~ 53 kDa) as a truncated form of CYP116B305. Tryptic digestion produced peptides spanning residues 24–461, primarily corresponding to the heme domain, with 40% sequence coverage and a 95% confidence interval. This truncated form co-purified with the desired holoenzyme across multiple chromatography steps, including affinity, size-exclusion, and anion-exchange chromatography (data not shown). Where relevant, the potential impact of the truncated form on the presented data has been considered.

**Fig. 3:**
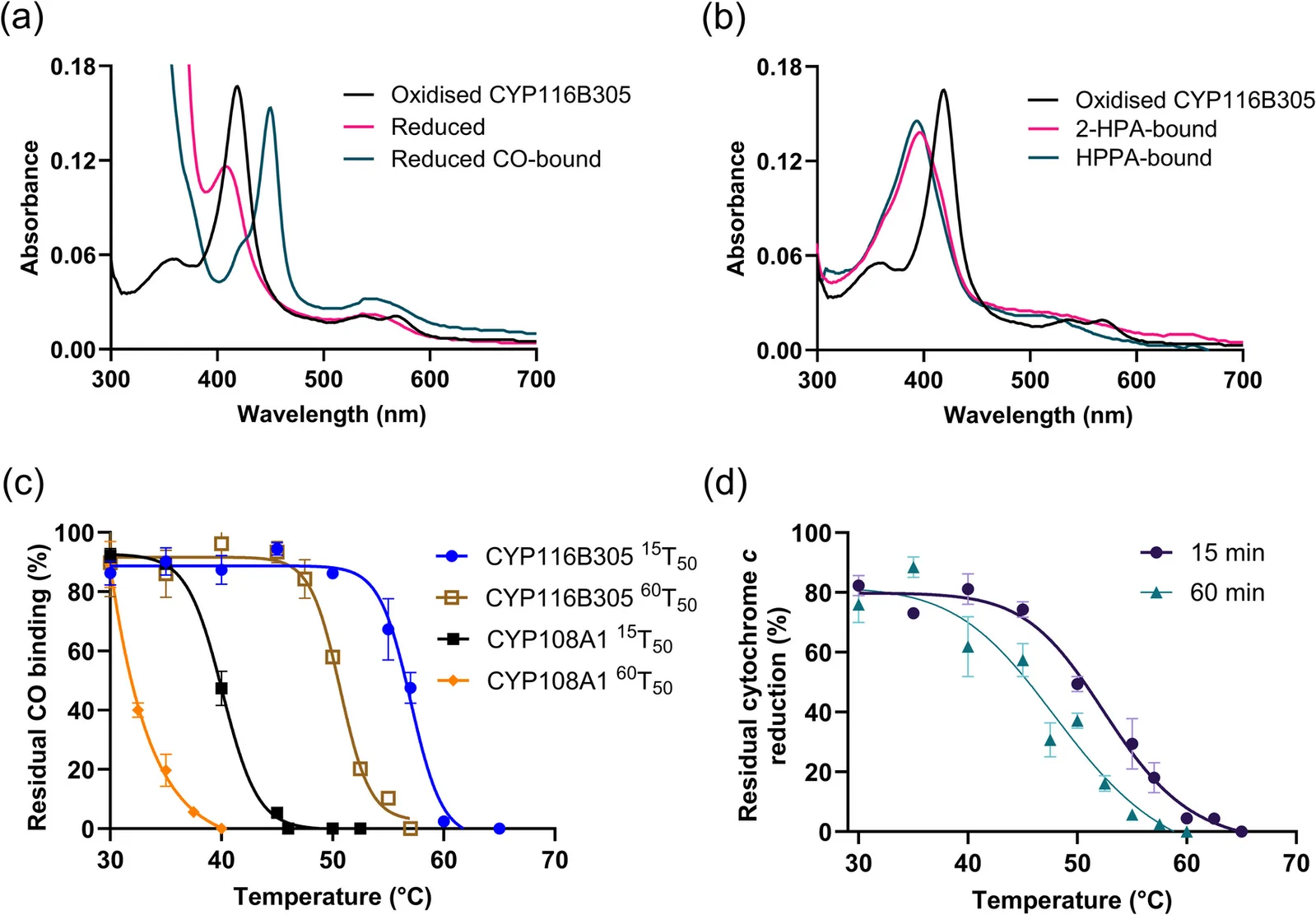
UV–visible spectra (300–700 nm) of purified CYP116B305: **a** oxidised form (black) has absorbance maxima at 418 nm, the reduced form at 410 nm (pink), and the reduced carbon monoxide complex form (green) at 450 nm and **b** oxidised CYP116B305 (black) in the presence of substrates 2-HPA (pink) and HPPA (green) has high-spin ferric heme absorbance peak at 396 nm. Thermal stability of CYP116B305 following the incubation of holoenzyme at elevated temperatures for 15 and 60 min: **c** plot of the residual heme content compared to CYP108 A1 and **d** plot of the residual cytochrome *c* reduction activity of CYP116B305 reductase domain. The obtained thermal stability data plots were fitted to a Hill-type equation (Eq. 1) using GraphPad Prism to extract *T*_50_ values

UV–visible absorbance spectroscopy of purified CYP116B305 (Fig. 3a) revealed the typical optical characteristics of an oxidised ferric P450, including the heme Soret peak at 418 nm and α-β bands at 569 nm and 536 nm, respectively. Upon reduction with sodium dithionite, the Soret peak shifted to 410 nm, and saturation with carbon monoxide resulted in the typical absorbance maxima at 450 nm, confirming the formation of the CO-bound ferrous P450 complex.

Based on sequence homology, CYP116B305 was predicted to contain FMN as its flavin prosthetic group, similar to other CYP116B enzymes. However, problems have long existed with the recombinant expression of self-sufficient P450 s, including low expression and incorporation of the prosthetic groups, especially the FMN cofactor (Hunter et al. 2005; Warman et al. 2012). To experimentally confirm the presence of FMN and quantify its content in the holoenzyme, the purified enzyme was subjected to heat denaturation to release the bound FMN (Chowdhary et al. 2007; Kim et al. 2018). The UV–visible spectra of released FMN in solution show two characteristic peaks at ∼375 nm and ∼445 nm, indicating a clear presence of an oxidised form of FMN cofactor (Fig. S4) (Macheroux 1999).

Subsequent quantification of free FMN (*ε*_445 nm_ = 12,500 M^−1^ cm^−1^) relative to heme (*ε*_450 nm_ = 91,000 M^−1^ cm^−1^) revealed that only 33% of CYP116B305 contained both heme and FMN cofactors. By comparison, the enzyme expressed without riboflavin supplementation in the culture medium showed an FMN content of only 20%, indicating that riboflavin addition improved FMN incorporation but was not significant enough to allow full incorporation in the holoenzyme. Similarly, co-expression with GroES/GroEL chaperones or increased riboflavin concentrations (150 µM) also failed to improve the FMN incorporation. This suboptimal cofactor (FMN) production likely contributed to the accumulation of truncated and immature polypeptides, as evidenced by the presence of truncated heme domain (Martínez-Limón et al. 2016).

To assess whether the truncated heme domain or holoprotein lacking the FMN retained its ability to receive electrons from the PFOR domain of the intact CYP116B305, NADPH-driven reduction followed by CO-difference spectroscopy was performed to measure formation of the CO-bound ferrous P450 complex (Fig. S5). This complex formation is only possible after the heme Fe^3+^ is reduced to Fe^2+^, and therefore, the proportion of CYP116B305 heme reduced by PFOR in the presence of NADPH was estimated and compared to sodium dithionite–reduced complex, assuming that the latter reduced 100% of the P450 heme. Interestingly, NADPH reduction produced 71% of the CO-bound signal intensity observed with sodium dithionite, despite the limited FMN occupancy (33%) in the intact holoenzyme.

### Thermal stability of the CYP116B305

The ability of self-sufficient CYP enzymes to transfer reducing equivalents from the reductase domain to the heme domain at elevated temperatures depends on the stability of both domains. Previous studies have indicated that the overall stability of these enzymes at high temperatures can be hindered by the limited thermostability of their reductase domains (Eiben et al. 2007; Tavanti et al. 2018b). Therefore, it is important to investigate the stability of both domains individually at higher temperatures.

Thus, the thermostability of the CYP116B305 was investigated through a two-part analysis. First, the stability of the heme domain was assessed by incubating the purified protein at elevated temperatures and measuring the residual heme concentration by CO-difference spectroscopy as described previously (Tavanti et al. 2018b; Harris et al. 2022). Since the CO-binding assay is purely concerned with the integrity of the heme domain, a cytochrome *c* assay was utilised to measure the relative stability of the reductase domain at higher temperatures (Guengerich et al. 2009; Tavanti et al. 2018b). The residual ferrous–CO complex formation (Fig. 3c) was determined using the CO-difference assay after incubation at various temperatures for either 15 or 60 min. From these data, the *T*_50_ value, representing the temperature at which 50% of intact P450 is retained, was calculated. For the heme domain of CYP116B305, the *T*_50_ after 15 min of incubation (^15^*T*_50_) was 56.9 ± 0.1 °C, while the *T*_50_ after 60 min of incubation (^60^*T*_50_) was 50.7 ± 0.1 °C. For comparison, the same values were measured for the mesophilic P450 CYP108 A1, yielding significantly lower *T*_50_ values of 40.1 ± 0.3 °C and 32.4 ± 0.5 °C after 15 and 60 min of incubation, respectively. Once again, no data was obtained that suggested a difference between the thermal stability of the holoenzyme and truncated heme domain.

Similarly, the thermal stability of the reductase domain was determined by measuring the residual cytochrome *c* activity after incubating the enzyme at temperatures ranging from 30 to 70 °C for 15 and 60 min (Fig. 3d). The *T*_50_ values were found to be 52.5 ± 0.5 °C for 15 min and 47 ± 1 °C for 60 min. It is also worth noting that the reductase domain retained only ~ 18% of the activity after 15 min of incubation at the previously determined heme domain ^15^*T*_50_ value of 57 °C, as assessed by a CO-binding assay. Interestingly, the reductase domain retained ~ 74% of the cytochrome *c* activity following incubation at 45 °C, which is the temperature at which the heme domain remained ~ 95% intact after 15 min.

### Binding studies

**Fig. 4:**
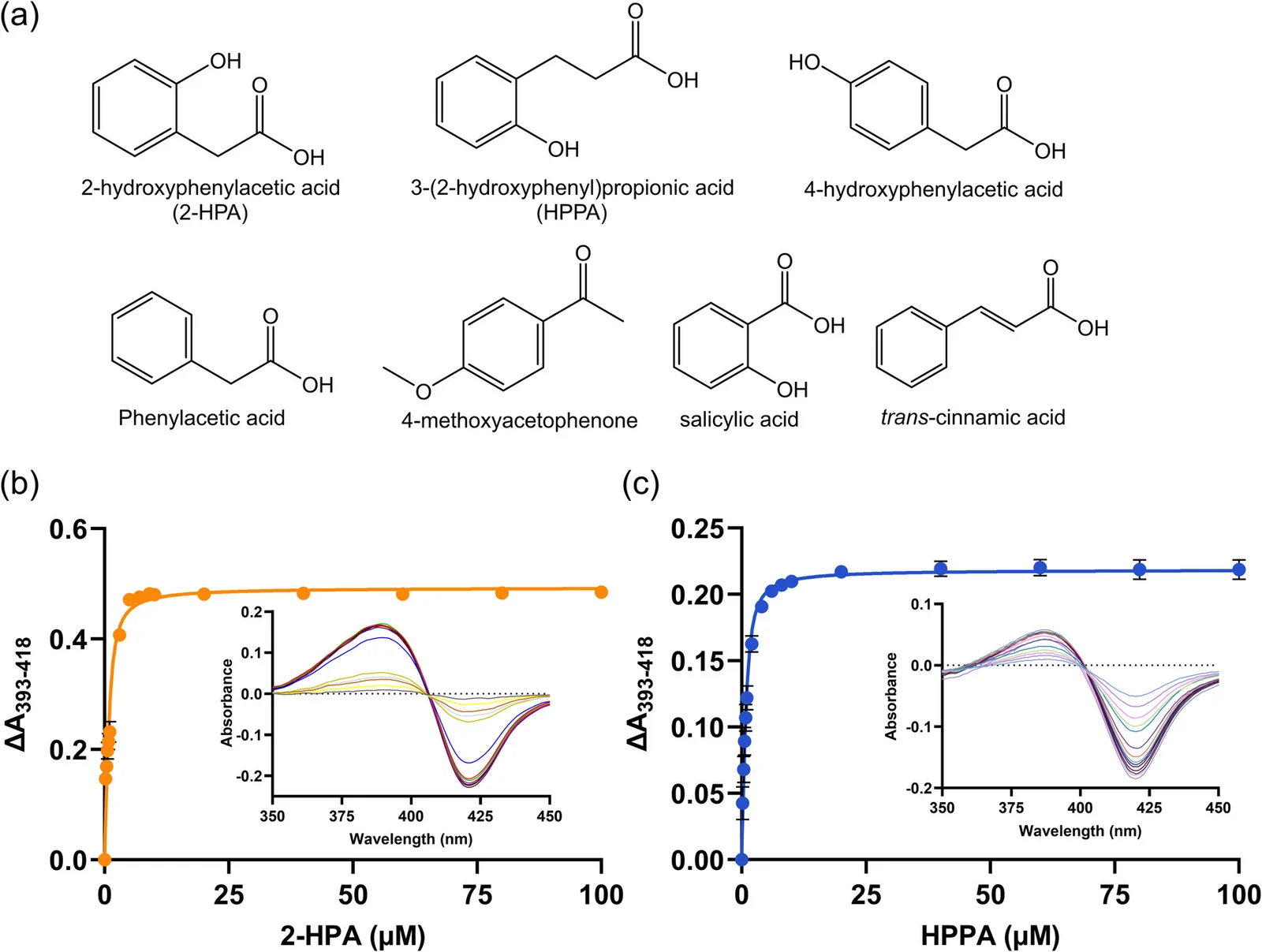
**a** Structures of compounds tested for binding with CYP116B305. Type I difference spectra and binding curves for **b** CYP116B305 (4 µM) with 2-HPA and **c** CYP116B305 (2 µM) with HPPA. Insets display the spectral shifts observed upon titration of CYP116B305 with substrates. The absorbance differences between the peak and trough in the spectral shifts were plotted against increasing substrate concentration to generate binding curves. The obtained data were fitted to tight-binding equation (Eq. 3) using GraphPad Prism to extract *K*_D_ values

A variety of aromatic substrates were tested for their ability to induce Type I spectral shifts upon binding to CYP116B305 (Fig. 4a). These shifts occur when a substrate displaces the water ligand from the heme iron, exhibiting a transition from the low-spin (~ 418 nm) to the high-spin (~ 390 nm) state, a characteristic feature of substrate binding in P450 enzymes. Two of the substituted aromatic compounds, 2-HPA and 3-(2-hydroxyphenyl)propionic acid (HPPA), gave the highest spin-state shifts (84 ± 3 and 90 ± 5%, respectively) (Fig. 3b) and bound the tightest (*K*_D_ = 0.4 ± 0.1 and 0.4 ± 0.2 µM, respectively) (Fig. 4b, c, Table 1). In the reduced state, the substrate-bound form of P450 displayed an absorbance maximum at 410 nm, similar to substrate-free reduction; however, the substrate-bound form showed a somewhat greater decrease in the Soret band (410 nm) intensity compared to the substrate-free form (Fig. S6).

**Table 1:** The dissociation constant (*K*_D_) and heme spin shift change of compounds investigated with CYP116B305 as potential substrates

Substrate	*K*_D_ (µM)	Spin-state shift (%)
3-(2-Hydroxyphenyl)propionic acid (HPPA)	0.4 ± 0.2 µM	90 ± 5%
2-Hydroxyphenylacetic acid (2-HPA)	0.4 ± 0.1 µM	84 ± 3%
Phenylacetic acid	39 ± 11 µM	34 ± 6%
4-Methoxyacetophenone	73 ± 6 µM	26 ± 3%
Salicylic acid	43 ± 6 µM	25 ± 2%
4-Hydroxyphenylacetic acid	65 ± 13 µM	17 ± 1%
*trans*-Cinnamic acid	61 ± 19 µM	13 ± 4%

Other structurally similar compounds (Table 1, Fig. S7) such as phenylacetic acid and *trans*-cinnamic acid, both of which lack a hydroxyl group, bound less tightly (*K*_D_ = 39 ± 11 and 61 ± 19 µM, respectively) to the P450 and gave a small spin shift change (34 ± 6 and 13 ± 4%, respectively), as compared to both the aforementioned tightly bound substrates. This highlights the importance of the presence of a hydroxyl group for tight binding to CYP116B305. Further, the significance of a hydroxyl group at the *ortho* position was suggested by the lower binding affinity of 4-hydroxyphenylacetic acid (*K*_D_ = 65 ± 13 µM) and its small induced spin-state shift (17 ± 1%). Moreover, salicylic acid, which carries an *o*-hydroxyl group but lacks an additional methylene moiety, also resulted in a lesser shift to high spin (25 ± 2%) as well as lesser binding affinity (*K*_D_ = 43 ± 6 µM). The influence of the presence of a methoxy group on the binding interaction with CYP116B305 was further analysed using 4-methoxyacetophenone, which also displayed a low binding affinity (*K*_D_ = 73 ± 6 µM) and induced a small spin-state shift (26 ± 3%).

**Fig. 5:**
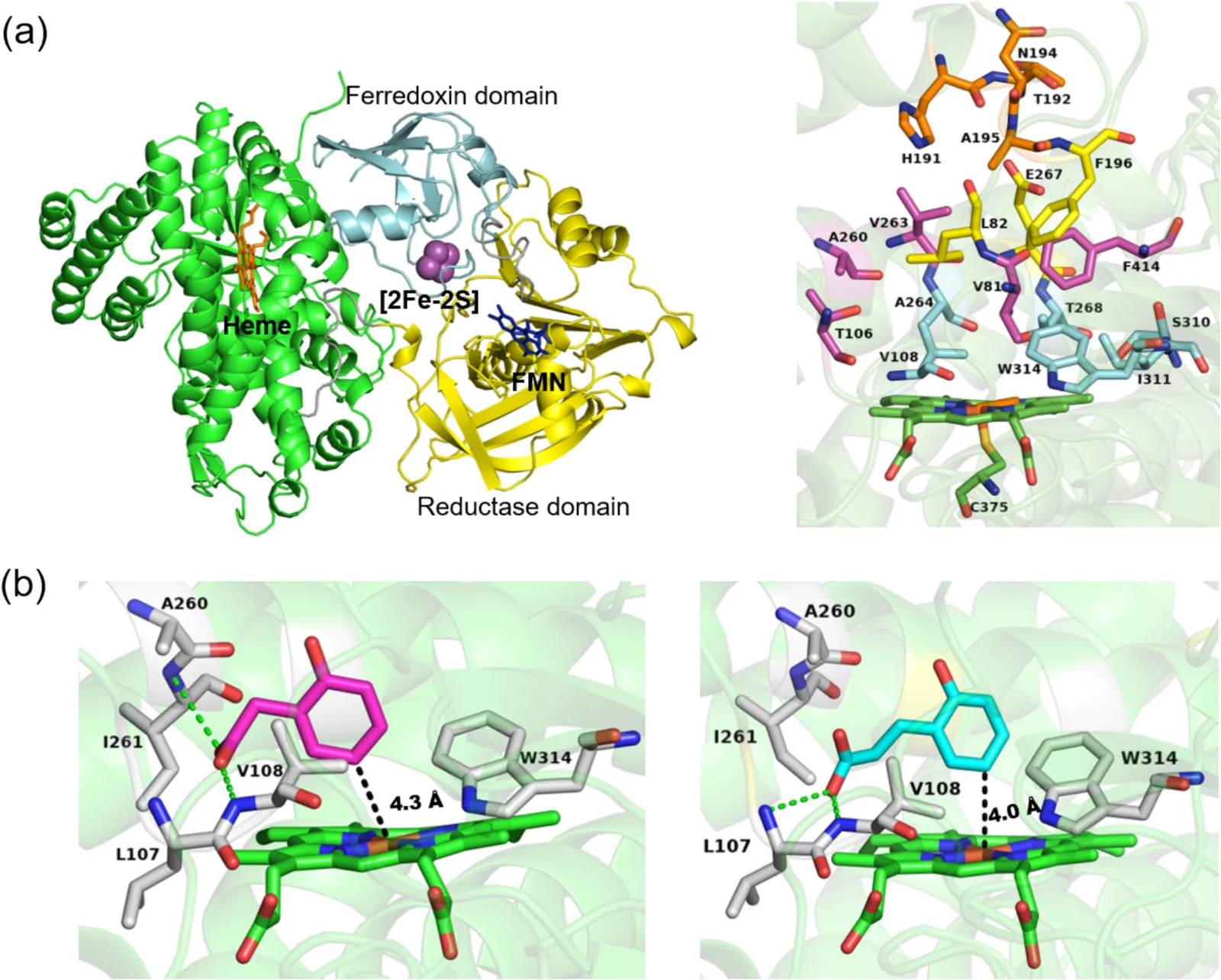
The predicted structure of CYP116B305 using AlphaFold 3 and visualized in PyMOL. **a** The full-length structure is presented as a cartoon showing heme, [2 Fe-2S], and FMN coloured in orange, magenta, and blue, respectively. Focused image on the right is showing the four tiers of active-site residues: tier 1 (cyan), tier 2 (magenta), tier 3 (yellow), and tier 4 (orange). **b** Environment of the active site in the 2-HPA (magenta) and HPPA (cyan) complexes with CYP116B305. Polar contacts are coloured in green

The crystal structures of CYP116B46 (Tavanti et al. 2018a) and CYP116B5 (Ciaramella et al. 2019) have enabled the visualisation and analysis of 18 residues lining the catalytic pocket, which likely interact with the substrate or are involved in the catalytic process. These active-site residues are categorised into four tiers above the heme and are generally conserved across CYP116B enzymes. A similar tiered arrangement and spatial positions of active-site residues were observed in the AlphaFold-predicted structure of CYP116B305 (Fig. 5a). Comparison with the thermophilic CYP116B46 revealed that most active-site residues in CYP116B305 are conserved, with the exception of two substitutions in tier 1: Pro320 and Val321 in CYP116B46 are replaced by Ser310 and Ile311, respectively, in CYP116B305 (Table S2). Similarly, comparisons with the mesophilic CYP116B234 showed broad conservation of active-site residues, with a few notable substitutions: Val314 (tier 1), Ala84 (tier 2), Trp199 (tier 3), and Thr198 (tier 4) in CYP116B234 correspond to Ile311, Val81, Phe196, and Ala195, respectively, in CYP116B305. Similar substitutions were noted when comparing CYP116B305 with other mesophilic CYP116B enzyme such as OhpA, mirroring the pattern observed with CYP116B234 (Table S2).

In silico modelling and docking studies were performed to further investigate substrate binding. Among the highest ranked poses, both substrates were found to orient their phenyl rings at position 5 approximately 4 Å from the heme iron centre, suggesting that substituted aromatic compounds are stably positioned within the catalytic pocket (Fig. 5b). These docking results align with previous observations, where CYP116B enzymes hydroxylate both substrates at the C5 position of their phenyl rings (Akter et al. 2024). For 2-HPA, key interactions included hydrophobic contacts between its phenyl ring and the side chains of Val108, Ala264, and Trp314, as well as interactions between its methylene moiety and the side chain of Ala260. Hydrogen bonding was predicted between the carboxylate group of 2-HPA and the backbone nitrogen atoms of Val108 (3.2 Å) and Ile261 (3.9 Å) (Table S3).

In contrast, for HPPA, hydrophobic interactions were observed between its phenyl ring and the side chains of Val108, Ala264, Trp314, and Phe414, as well as interactions between its methylene moieties bonded to the benzene ring and the side chain of Val108. Furthermore, hydrogen bonds were formed between the carboxylate moiety of the propionic acid chain of HPPA and the backbone nitrogen atoms of Leu107 (3.6 Å) and Val108 (3.1 Å) (Table S3).

Although Leu107 and Ile261 fall outside the designated tiered system, they emerged as important contributors to hydrogen bonding with substrates. However, these residues exhibit variability across CYP116B enzymes, with Leu107 often substituted by methionine and Ile261 by glycine. These variations may influence substrate specificity or catalytic efficiency, thereby highlighting the need for further investigation.

### Cofactor preference and kinetics

In order to determine the nucleotide cofactor preference for CYP116B305, steady-state kinetic experiments were performed to determine the rate of oxidation of NADPH and NADH by CYP116B305 in the presence of the substrate 2-HPA at 25 °C. Nonlinear regression analysis was used to derive *K*_*M*_ and *k*_cat_ values for both the nicotinamide cofactors. While CYP116B305 was observed to accept electrons from both NAD(P)H cofactors, NADPH displayed a 15-fold lower *K*_*M*_ (4.1 ± 0.3 µM) compared to NADH (60 ± 15 µM) (Fig. S8). Although NADH showed a higher turnover number (*k*_cat_ = 39 ± 4 min^−1^ vs 13 ± 1 min^−1^ for NADPH), the catalytic efficiency (*k*_cat_/*K*_*M*_) favoured NADPH by approximately fivefold (3 ± 1 µM^−1^ min^−1^ vs 0.65 ± 0.12 µM^−1^ min^−1^ for NADH), revealing a strong preference for NADPH over NADH as an electron donor. Further kinetic analysis using cytochrome *c* as an artificial electron acceptor and NADPH as the electron donor gave similar *K*_*M*_ and *k*_cat_ values for NADPH, measured as 1.2 ± 0.1 µM and 4.1 ± 0.1 min^−1^, respectively (Fig. S9).

Furthermore, to get insight into the temperature-dependent oxidation of NAD(P)H by substrate-bound CYP116B305, nicotinamide cofactor oxidation rates were measured at 45 °C and compared to those at 25 °C (Table 2). For 2-HPA, the NADPH oxidation rates increased by 6.5-fold, from 13 ± 1 min^−1^ at 25 °C to 85 ± 3 min^−1^ at 45 °C. Similarly, with HPPA, a sixfold increase in NADPH oxidation from 11 ± 1 to 65 ± 3 min^−1^ was observed. A comparable trend was observed for NADH oxidation, with rates increasing 3.2-fold in the presence of 2-HPA (26 ± 2 to 83 ± 10 min^−1^) and 2.4-fold with HPPA (31 ± 3 to 73 ± 2 min^−1^).

**Table 2:** Activity parameters determined for substrates investigated with CYP116B305. Shown are NAD(P)H consumption rates and coupling efficiency measurements at 25 °C and 45 °C. Rates are µM (µM^−1^ P450) min^−1^ (abbreviated as min^−1^ in the text). n.q. refers to non-quantifiable. n.d. refers to no product observed/no NADPH oxidation

Substrate	T (°C)	NADPH consumption rates (100% FMN rates)	NADH consumption rates (100% FMN rates)	Coupling (NADPH)	Coupling (NADH)
2-HPA	25 °C	13 ± 1 (~ 39)	26 ± 2 (~ 79)	60 ± 18%	31 ± 3%
	45 °C	85 ± 3 (~ 257)	83 ± 10 (~ 251)	82 ± 4%	58 ± 3%
HPPA	25 °C	11 ± 1 (~ 33)	31 ± 3 (~ 100)	15 ± 2%	~ 5%
	45 °C	65 ± 3 (~ 196)	73 ± 2 (~ 221)	26 ± 1%	15 ± 9%
4-Methoxy acetophenone	25 °C	n.d	2 ± 1 (~ 6)	n.q	n.d
	45 °C	1 ± 1 (~ 3)	3 ± 1 (~ 9)	24 ± 5%	n.d

Of the substrates with low binding affinity with CYP116B305, only 4-methoxyacetophenone stimulated the oxidation of NAD(P)H, albeit a very low rate (Table 2). Notably, the above oxidation rates correspond to 33% FMN inclusion relative to the heme, and therefore, the values corresponding to the full incorporation of FMN relative to heme are also indicated in the table as an approximate maximum activity value. No NADPH oxidation above the background rate was observed for all the other more weakly bound substrates mentioned in Table 1.

### In vitro analysis of CYP116B305 and coupling efficiency measurements

The above measurements focused only on determining the rate of NAD(P)H consumption. However, substrate and cofactor identification rely on two key factors: first, the formation and identification of the reaction products and second, the efficiency of electron transfer from the cofactor NAD(P)H to the substrate during oxidation. To address this, we tested the in vitro oxidation of all substrates previously assessed for binding, using both nicotinamide cofactors. GC–MS analysis of the BSTFA-TMS derivatised turnover extracts was used to determine the formation and identification of products based on the mass fragmentation pattern as compared to either authentic standards or a GC–MS spectrometric library (Wiley, NIST).

**Fig. 6:**
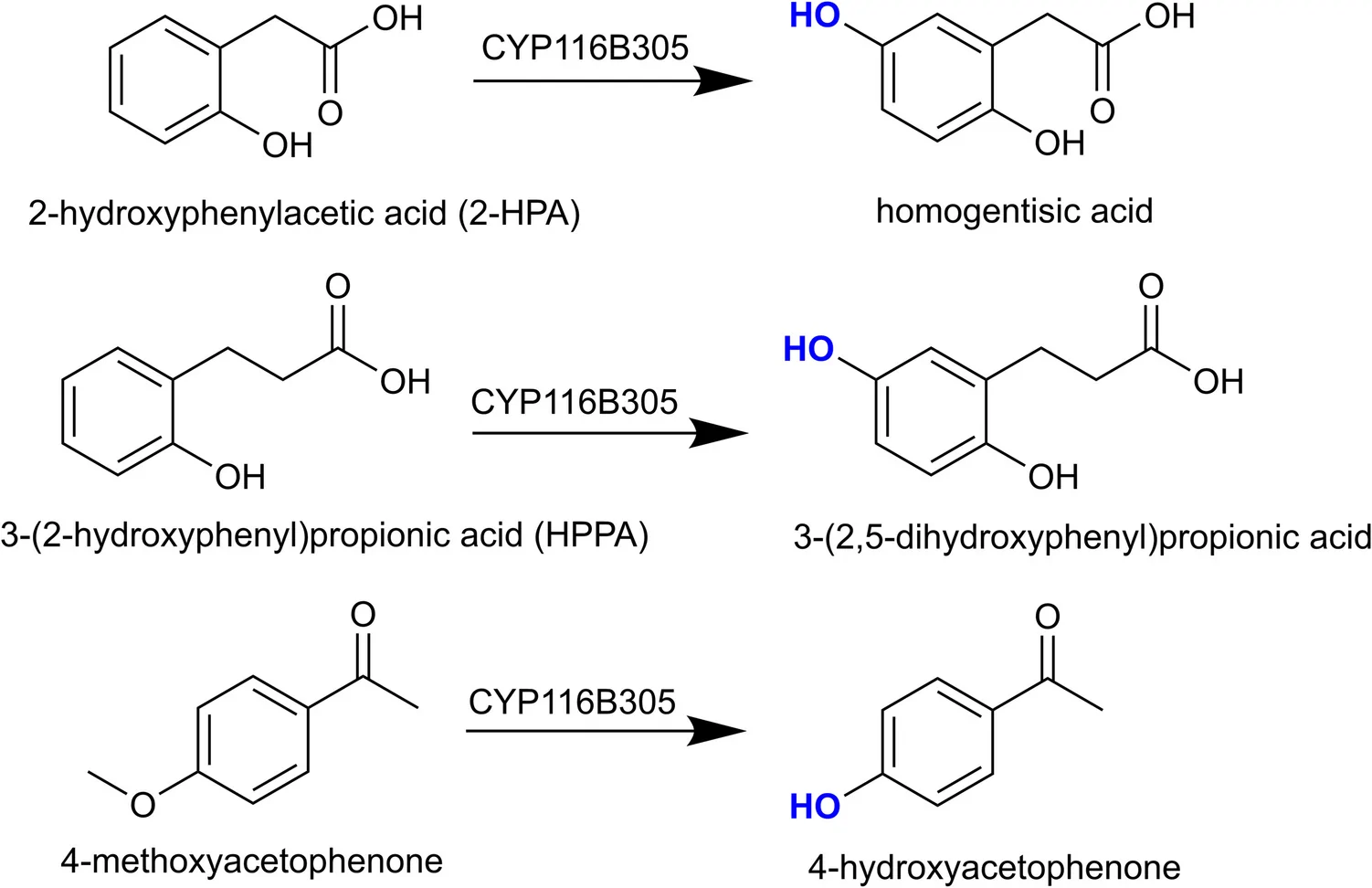
In vitro reactions catalysed by the CYP116B305 enzyme

**Fig. 7:**
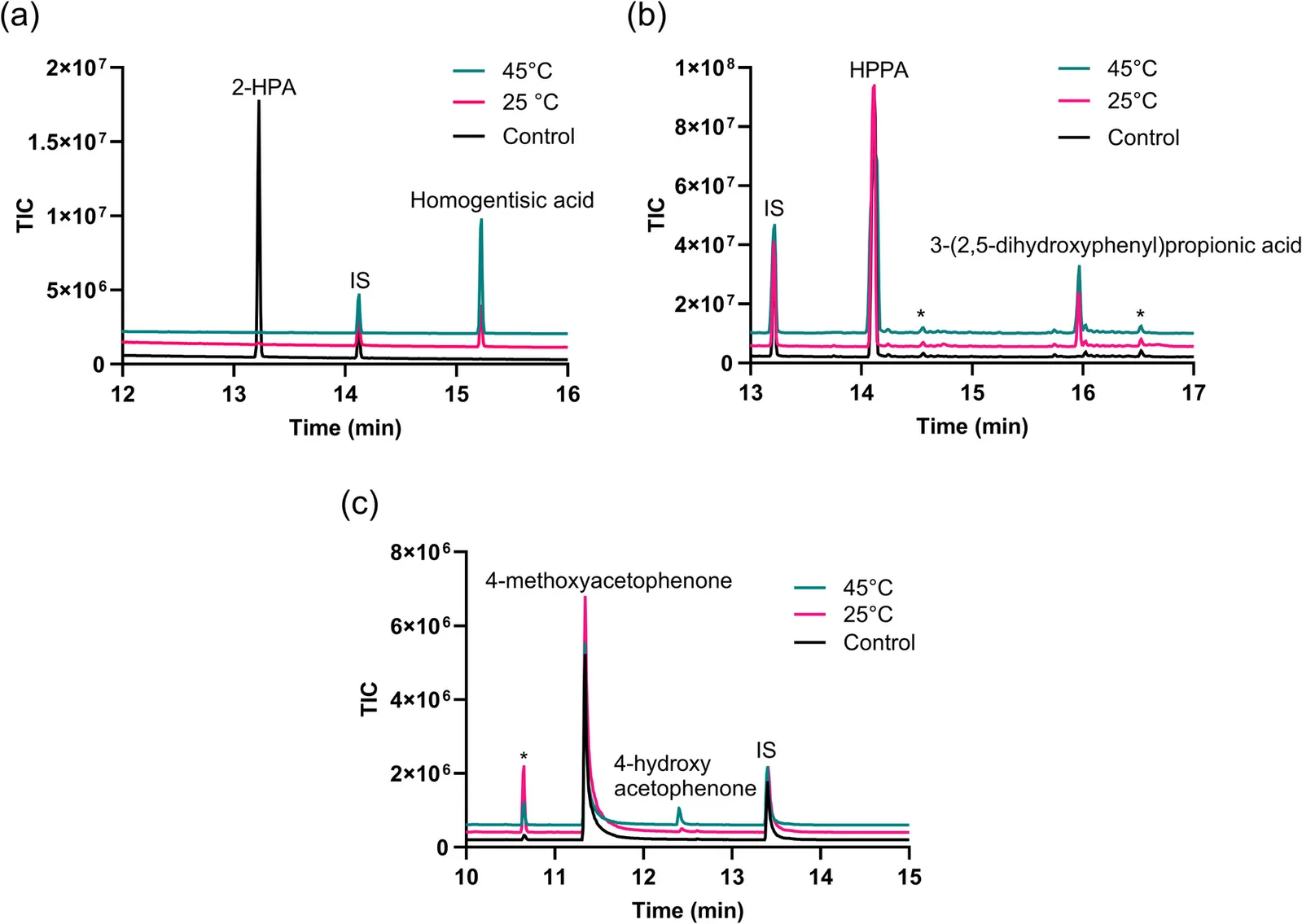
GC chromatogram of products following in vitro NADPH oxidation of substrates **a** 2-HPA, **b** HPPA, and **c** 4-methoxyactophenone. The internal standard (IS) is as indicated. Products were extracted into ethyl acetate and derivatised using BSTFA-TMS before GC–MS analysis. Other peaks marked as stars (*) are various organosilicon contaminants with mass fragmentations patterns unrelated to the substrate or product

The self-sufficient system of CYP116B305 was tested for its ability to catalyse product formation at both room temperature (~ 25 °C) and elevated temperature (45 °C). Under both conditions, CYP116B305 catalysed the hydroxylation of 2-HPA at the C5 position, generating 2,5-dihydroxyphenylacetic acid (homogentisic acid) in the presence of either NADPH or NADH (Fig. 6). The mass spectrum of the BSTFA-TMS derivatised product exhibited an expected molecular ion peak at *m*/*z* = 384, along with a fragmentation pattern and retention time consistent with the TMS-derivatised authentic standard of homogentisic acid (Fig. S10). Notably, when NADPH was supplied in a stoichiometric ratio to the substrate, complete consumption of 2-HPA was observed at both 25 °C and 45 °C (Fig. 7a). However, in the presence of NADH, complete conversion of 2-HPA was not achieved at either temperature (Fig. S10).

Similarly, CYP116B305 catalysed hydroxylation of HPPA at the C5 position, producing 3-(2,5-dihydroxyphenyl)propionic acid in the presence of either nicotinamide cofactor at both 25 °C and 45 °C (Figs. 6 and 7b). The product was identified through its unique mass fragmentation profile which matched that reported in the literature for TMS-derivatised 3-(2,5-dihydroxyphenyl)propionic acid (Fig. S11) (Heindl et al. 1985; Kundral et al. 2025).

We also investigated the in vitro oxidation of 4-methoxyacetophenone by CYP116B305. The enzyme catalysed demethylation at the C4 position, producing 4-hydroxyacetophenone in the presence of NADPH at both 25 °C and 45 °C (Figs. 6 and 7c, Fig. S12), but no product was observed when NADH was used as the electron donor (data not shown). In contrast to 2-HPA, the oxidation of HPPA and 4-methoxyacetophenone resulted in incomplete substrate consumption and significantly lower product yields under all tested conditions (Table 2, Fig. 7). No enzymatic product was observed for any of the other compounds in Table 1.

Further experiments were conducted to determine the coupling efficiency of CYP116B305-catalysed oxidation reactions by measuring the ratio of the product formed to the NAD(P)H consumed. The inefficient transfer of reducing equivalents from NAD(P)H to P450 heme leads to the formation of reactive oxygen species (ROS) as the side products, a process commonly known as “uncoupling”.

At 25 °C, CYP116B305-catalysed oxidation of 2-HPA exhibited a coupling efficiency of 60 ± 18% with NADPH, whereas HPPA oxidation was significantly lower at only 15 ± 2%. Coupling efficiency assays at 45 °C showed an improvement, with coupling efficiency increasing to 82 ± 4% for 2-HPA and 26 ± 1% for HPPA. Previous NADPH consumption assays revealed a significant increase in electron transfer rate at 45 °C compared to 25 °C (Table 2). This increase in the electron transfer rate and coupling efficiency highlights the improved catalytic performance of CYP116B305 at elevated temperatures.

It was also observed that homogentisic acid undergoes polymerisation, evidenced by a red colouration of the solution after oxidation of 2-HPA. Similar catalytic activity has been observed in other CYP116B enzymes which tentatively identified the red colour pigment as pyomelanin, formed through the auto-oxidation and polymerisation of homogentisic acid (Donoso et al. 2021; Akter et al. 2024). Thus, the quantification of the homogentisic acid peak using GC–MS was not a reliable measure of product formation. Therefore, the coupling efficiency of CYP116B305-catalysed hydroxylation of 2-HPA to homogentisic acid was determined by substrate depletion rather than product formation.

For 4-methoxyacetophenone, NADPH oxidation rates were significantly low at both temperatures. Oxidation at 25 °C yielded only a small amount of product while oxidation at 45 °C resulted in sufficient product formation to determine a coupling efficiency of 24 ± 5%.

Further measurements of electron coupling efficiency in CYP116B305-catalysed oxidation reactions in the presence of NADH were undertaken (Table 2). We compared the reaction at 25 °C and 45 °C. At 25 °C, coupling efficiency for 2-HPA oxidation was found to be 31 ± 3%, while HPPA oxidation showed a negligible product formation, corresponding to an estimated coupling efficiency of approximately 5%. Despite a higher electron transfer rate with NADH compared to NADPH for both the substrates, the coupling efficiency remained lower than that observed with NADPH.

At 45 °C, NADH-dependent oxidation by CYP116B305 showed an improved coupling efficiency of 58 ± 3% for 2-HPA, while HPPA oxidation reached only 15 ± 9%. Although the electron transfer rate for both nicotinamide cofactors was comparable at 45 °C, NADH coupling efficiency remained significantly lower than that observed with NADPH. These findings again suggest that CYP116B305 can utilise both nicotinamide cofactors, but the enzyme displays a clear preference for NADPH.

### Redox characterisation

Optical spectroelectrochemistry was employed to investigate the redox characteristics of the heme and PFOR domains of CYP116B305. The determinations of redox potentials for such enzymes can be challenging due to overlapping spectral contributions from each cofactor. Nonetheless, two distinct phases of reduction are often observed for self-sufficient P450 enzymes. The first phase involves reduction of the FMN-Fe-S domain in the range of 0 to − 200 mV vs NHE, with major spectral changes observed between 450 and 500 nm, where interference from the heme is negligible. The second phase corresponds to the reduction of the heme, often evidenced by a shift in the Soret peak to ~ 410 nm, which occurs in the approximate range of − 200 to − 500 mV.

**Fig. 8:**
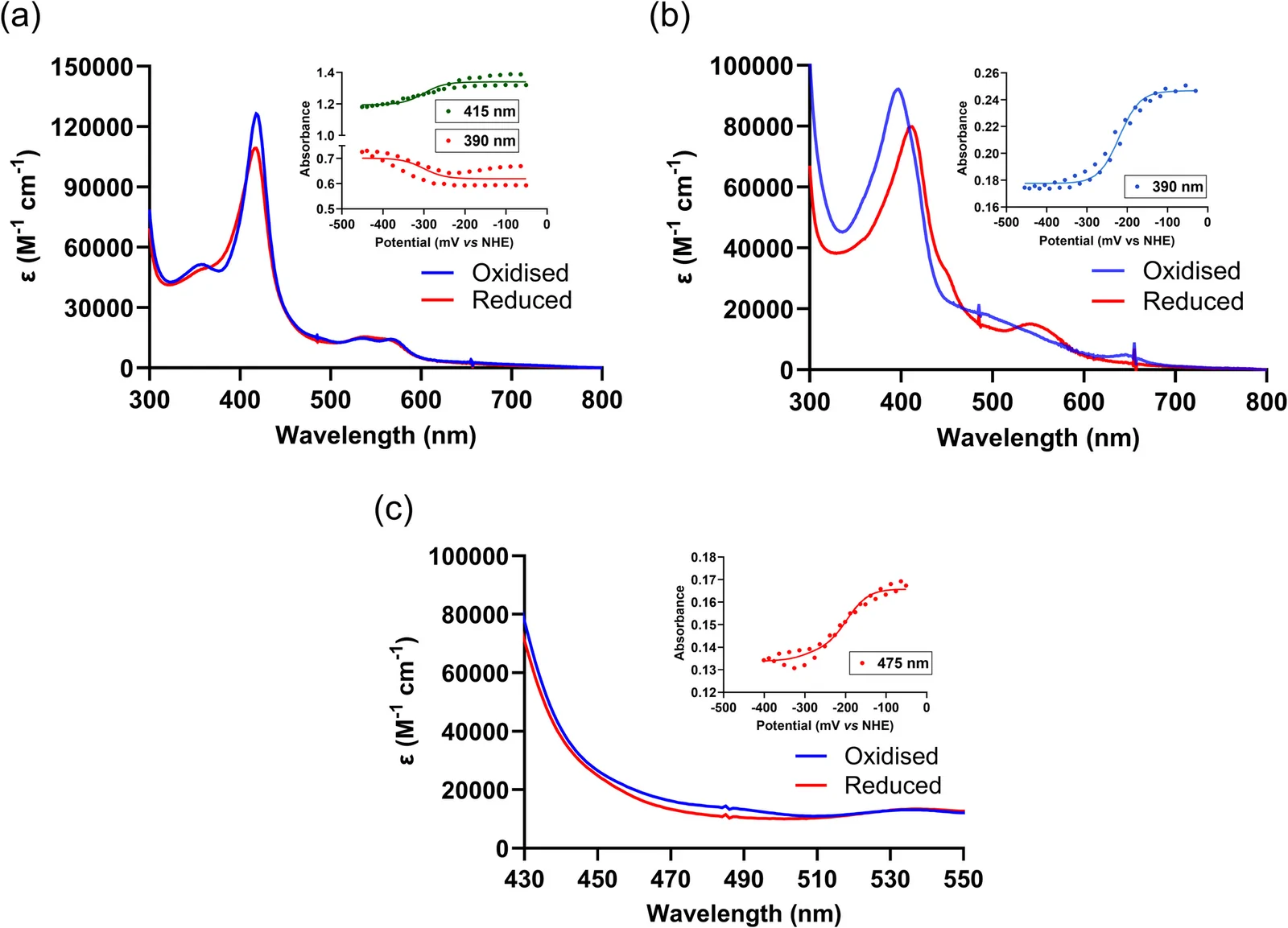
UV–visible spectra of spectroelectrochemical titration of **a** substrate-free CYP116B305 (66 µM) at 35 °C, **b** substrate-bound (100 µM 2-HPA) CYP116B305 (17 µM) at 20 °C showing both oxidised (blue) and reduced (red) states, and **c** substrate-free CYP116B305 (66 µM) at 20 °C demonstrating the reduction of the FMN and [2 Fe-2S] clusters. Inset*s display* selected single wavelength absorption values due to applied potential

In the substrate-free form of CYP116B305, electrochemical reduction did not result in the expected Soret band shift to 410 nm, as seen with chemical reduction (Fig. S6). Instead, the Soret band slightly decreased in intensity without distinct changes in the α-β region, which is a phenomenon also observed previously in other P450 s such as CYP124 A1 (Mohamed et al. 2023) and CYP116B234 (Kundral et al. 2025). Experiments at 20 °C showed significant hysteresis in the region associated with heme (Fig. S13), whereas similar measurements at 35 °C produced reversible spectral changes with significantly less hysteresis (Fig. 8a). The spectral changes in the heme Soret band (415 nm) and α-β bands (555 nm) were analysed as a function applied potentials as these regions do not overlap significantly with absorbances from the FMN/Fe-S cofactors. A mid-point potential of − 300 mV vs NHE was calculated for the heme in the substrate-free CYP116B305.

By contrast, spectroelectrochemical experiments of 2-HPA-bound CYP116B305 mirrored the chemical reduction results, showing the characteristic 410 nm peak associated with the fully reduced P450 (Fig. 8b). Minimal hysteresis associated with relatively much larger reversible spectral changes was observed in these titrations with the original ferric heme spectrum recovered upon oxidation. Data were collected over the whole spectral range (300–800 nm), but only spectral changes of the heme Soret region (300–450 nm) were analysed to remove any spectral interference from the FMN/Fe-S domains. This resulted in the calculated heme redox potential of − 220 mV vs NHE for 2-HPA-bound CYP116B305. Furthermore, no data indicated any influence of the truncated heme domain on the redox potential measurements.

Finally, the reduction of the FMN and [2 Fe-2S] centres (PFOR domain) of CYP116B305 was investigated by analysing spectral changes in the 430–550 nm region. In the substrate-bound form, the redox potentials of the heme and PFOR domain significantly overlapped and therefore could not be determined independently. However, in the substrate-free form at 20 °C, distinct spectral changes in the 430–550 nm region typically associated with a PFOR domain were observed with minimal hysteresis. Assuming that spectral changes in this region are dominated by the FMN chromophore while the Fe-S centre is much weaker (Harmer et al. 2023), the calculated potentials correspond to the two redox reactions of the flavin. Thus, spectral analysis provided calculated reduction potentials of − 155 and − 250 mV vs NHE (Fig. 8c). While heme-associated spectral changes at 35 °C were distinct, no significant shifts were observed in the PFOR domain along with substantial hysteresis, likely due to stability issues associated with the PFOR domain at elevated temperatures.

## Discussion

The limited thermostability of existing P450 biocatalysts has long been a barrier to their industrial applications, and the discovery of new thermostable P450 enzymes is time-consuming due to the lack of cultured isolates. Metagenomic sequencing offers a promising alternative by enabling the discovery of predicted novel enzymes directly from environmental samples, bypassing the need for cultivation. Despite this potential, only three bacterial P450 enzymes (SYK181, P450-T2, and P450-T3) have been identified through metagenomic approaches (Kim et al. 2007; Nguyen et al. 2020, 2021). By leveraging sequence-based metagenomics, this study has identified and characterised a previously unreported thermophilic cytochrome P450 enzyme, CYP116B305, from an uncultivated, low abundance microorganism present in hot springs from Northern Australia. CYP116B305 shares the highest amino acid sequence identity (68%) to the known thermostable CYP116B46 from *T. thermophilus*. The recombinant CYP116B305 protein was produced in *E. coli* (~ 200 nmol L^−1^ of culture) and purified using standard procedures; however, only 33% of the hemoprotein incorporated FMN. Similar variation in FMN-to-heme ratios has been reported for other CYP116 enzymes, including ∼55% for CYP116B1 (Warman et al. 2012), 27–100% for CYP116B2 (Hunter et al. 2005), and 75% for CYP116B234 (Kundral et al. 2025). The relatively low FMN content observed in CYP116B305 suggests that the heterologous production conditions for flavin incorporation are suboptimal leading to truncation and/or the flavin cofactor is more weakly bound, resulting in significant loss during the purification process.

UV–visible spectroscopy of the purified protein did not reveal distinct Soret peaks that would indicate separate populations of holoenzyme and truncated heme domain, suggesting that the heme environment remains consistent, despite partial FMN occupancy. NADPH-driven reduction showed that the truncated heme domain of CYP116B305 retained efficient electron transfer from the PFOR domain, achieving 71% heme reduction despite only 33% FMN occupancy, and SDS-PAGE analysis indicated the presence of a significant amount of truncated (heme domain only) protein. This finding demonstrates an intermolecular electron transfer from the PFOR domain of the intact holoenzyme to its cognate heme domain and to other proteins, the truncated heme domain and/or the full-length protein lacking the FMN.

Stability assays demonstrated thermostability of CYP116B305, highly comparable to the structurally similar and thermostable CYP116B46 (Tavanti et al. 2018b). The ^15^*T*_50_ value for the heme domain of CYP116B305 (56.9 ± 0.1 °C) was found to be approximately 17 °C higher than that of the mesophilic enzyme CYP108 A1 (Peterson et al. 1992). A similar trend was observed for the ^60^*T*_50_ values, with CYP116B305 showing an increase of approximately 18 °C (50.7 ± 0.1 °C) when compared to that of CYP108 A1. Notably, CYP116B305 lost very little integrity when incubated at temperatures between 30 and 45 °C for 15 min; hence, 45 °C was selected as the optimal temperature for further experiments assessing the enzyme’s temperature-dependent activity. A small disparity in the thermal stability between the heme (56.9 ± 0.1 °C) and the reductase domain (52.5 ± 0.5 °C) of CYP116B305 was observed; however, both demonstrated significant thermal stability. Thermophilic P450 enzymes in the CYP116B subfamily exhibit *T*_50_ values between 42 and 60 °C (Tavanti et al. 2018b). In contrast, other thermophilic P450 enzymes, primarily from archaeal sources, can have *T*_50_ values reaching up to 90 °C, classifying them as hyperthermophiles (McLean et al. 1998; Oku et al. 2004). The *T*_50_ value for CYP116B305 (heme) is 56.9 ± 0.1 °C, placing it within the typical range for CYP116B enzymes and categorising it as a moderately thermophilic enzyme.

The substrate binding profile of CYP116B305 closely aligns with that of two other CYP116B enzymes, the mesophilic CYP116B234 (Kundral et al. 2025) and the thermophilic CYP116B46 (Akter et al. 2024). However, differences in binding affinities and heme spin-state shifts were observed. For instance, 2-HPA exhibits tight binding with all three enzymes, but variations in heme spin-state shifts are significant, with CYP116B234 showing a 66% shift (Kundral et al. 2025) and CYP116B46 showing only a 25% shift (Akter et al. 2024). Moreover, both CYP116B234 and CYP116B46 have shown reduced binding affinities for HPPA compared to 2-HPA. In contrast, CYP116B305 exhibits comparably high heme spin-state shifts with both 2-HPA and HPPA, along with similar high binding affinities for these substrates, marking a distinct characteristic of this enzyme. Overall, all three enzymes display the strongest binding affinity for benzoic acid substrates with *ortho*-hydroxyl groups (e.g. 2-HPA, HPPA). Furthermore, the large spin-state shift observed with HPPA across all enzymes highlights the importance of substrate side chain length in protein interactions.

Given the structural similarity and overlapping substrate binding profiles within the CYP116B family, analysing the active-site residues of P450 enzymes is critical to understanding their substrate selectively and specificity. The observed substitutions in CYP116B305 relative to mesophilic and thermophilic homologs do not appear to significantly alter substrate preference, supporting the idea that members of the CYP116B family likely share the same core substrate(s). Furthermore, in silico modelling and docking studies revealed various hydrophobic and hydrogen bonding interactions of the side chain and phenyl ring of both the substrates with the active-site residues of CYP116B305. However, despite the binding affinity evidence demonstrating the critical importance of the *ortho*-hydroxyl group in the substrates for tight binding to CYP116B305, these docking studies did not reveal significant interactions between this group and active-site residues. This suggests that water molecules may mediate these interactions, highlighting the need for experimental structure determination to better understand the enzyme–substrate interactions.

The preference of CYP116B305 for NADPH over NADH is consistent with other CYP116B enzymes where NADPH typically exhibits a *K*_*M*_ value that is 3–500-fold lower than that of NADH (Roberts et al. 2003; Warman et al. 2012; Yin et al. 2014; Tavanti et al. 2018b; Porter et al. 2018; Correddu et al. 2023). Moreover, the higher NAD(P)H oxidation rates observed with 2-HPA and HPPA over other structurally similar substrates highlight the selectivity of CYP116B305 enzyme for these substrates. Furthermore, the improved NADPH consumption rates at elevated temperature (45 °C) in comparison to 25 °C align with that reported for the related enzyme, CYP116B46. The latter demonstrated more than a twofold increase in NADPH oxidation rates at 50 °C compared to 30 °C for both substrates, while its NADH oxidation rates showed only a marginal increase (Akter et al. 2024). Notably, while CYP116B305 exhibited significantly lower NADPH oxidation rates than CYP116B46, its NADH oxidation rates were highly similar to those observed for CYP116B46.

Despite HPPA inducing a comparable heme spin-state shift and exhibiting an equally high binding affinity, CYP116B305 demonstrated a higher NADPH coupling efficiency and complete substrate conversion with 2-HPA. This selective preference for 2-HPA mirrors observations in CYP116B46 and CYP116B234, which similarly favour 2-HPA despite a higher spin-state shift and tighter binding being observed with HPPA. This trend further supports the proposed physiological role of CYP116B enzymes in initiating the homogentisate pathway (Donoso et al. 2021; Kundral et al. 2025) and highlights the role of CYP116B305 from Geminococcaceae in catalysing the oxidation of such organic acids, particularly 2-HPA. While CYP116B enzymes, including CYP116B305, exhibit catalytic promiscuity, as evidenced by its catalysis of NADPH-dependent demethylation of 4-methoxyacetophenone, their efficiency significantly declines for such non-physiological substrates. Interestingly, CYP116B305 exhibited a consistent improvement in coupling efficiency at elevated temperatures, correlating with an enhanced electron transfer rate. In contrast, CYP116B46 reported either similar or reduced coupling efficiency at higher temperatures, despite increased electron transfer rates (Akter et al. 2024).

The redox potential of the heme domain in substrate-free CYP116B305 (− 300 mV vs NHE) closely aligns with the value reported for the intact CYP116B1 (− 301 ± 7 mV vs NHE) (Warman et al. 2012). However, studies on other CYP116B enzymes, such as P450 RhF and CYP116B234, in their substrate-free forms have generally reported more negative redox potentials, often around − 400 mV vs NHE (Hunter et al. 2005; Kundral et al. 2025). Upon substrate (2-HPA) binding, the heme redox potential of CYP116B305 exhibits a redox potential shift of + 80 to − 220 mV vs NHE. This facilitates electron transfer from the PFOR domain, the FMN of which was found to have redox potentials of − 155 and − 250 mV vs NHE by spectroelectrochemistry. This shift in heme redox potential upon substrate binding, while significant, is relatively moderate compared to that observed with CYP116B234 (+ 160 mV), which also has a more negative potential for substrate-bound heme (− 270 mV). The reported redox potentials for PFOR domain in CYP116B234 are − 240 mV for FMN and − 212 mV for [2 Fe-2S] cluster (Kundral et al. 2025). Despite these findings, overlapping redox transitions of FMN and Fe-S clusters in the intact enzyme complicate their independent resolution, emphasising the need to study these domains in isolation. Future efforts will therefore focus on dissecting the individual domains to better understand their redox characteristics.

Overall, the improved NADPH consumption rates and coupling efficiency measurements at elevated temperatures highlight its potential for biotechnological applications under higher temperature conditions. Moving forward, research will focus on optimising FMN incorporation into the full-length holoenzyme, further improving the reductase domain’s stability, and broadening substrate scope through protein engineering. The observed evidence for possible intermolecular electron transfer between an intact holoenzyme’s PFOR and the heme domain will motivate future studies of an isolated PFOR domain. Together, these efforts will enhance CYP116B305’s performance and adaptability for large-scale biotransformations.

## Supplementary Information

Below is the link to the electronic supplementary material.

ESM 1 (DOCX 5.21 MB)

## Data Availability

This metagenomes have been submitted to ENA under the project accession number PRJEB88793.
