# Supplementary material for: Characterisation of the thermophilic P450 CYP116B305 identified using metagenomics-derived sequence data from an Australian hot spring
Source: Appl Microbiol Biotechnol. 2025 May 31;109:133. doi: 10.1007/s00253-025-13521-2 (PMC12126322; doi:10.1007/s00253-025-13521-2)
Supplement: Supplementary file 1 [file 253_2025_13521_MOESM1_ESM.docx]

**Supporting Information**

**Characterisation of the thermophilic P450 CYP116B305 identified using Metagenomics-Derived Sequence Data from an Australian hot-spring**

*Simran Kundral ^a b c^, Peter D. Giang ^b^, Leah R. Grundon ^d^, Jenna M. Supper ^d^, Sunil K. Khare ^c e^, Paul V. Bernhardt ^b^, Paul Evans ^d^, Stephen G. Bell ^f^, James J. De Voss ^b*^*

*^a^ The University of Queensland - Indian Institute of Technology Delhi Research Academy (UQIDRA), India*

*^b^ School of Chemistry and Molecular Biosciences, The University of Queensland, Brisbane 4072, Australia*

*^c^ Enzyme and Microbial Biochemistry Laboratory, Department of Chemistry, Indian Institute of Technology Delhi, India*

*^d^ Australian Centre for Ecogenomics, School of Chemistry and Molecular Biosciences, University of Queensland, Brisbane 4072, Australia*

*^e^ Department of Biological Sciences, Indian Institute of Science Education and Research Kolkata, India*

*^f^ Department of Chemistry, The University of Adelaide, Adelaide, SA, 5005, Australia.*

Contents

[CYP116B305 protein sequence	3](#_Toc192689920)

[Protein Composition and Stability	3](#_Toc192689921)

[Amino acid sequence alignment of CYP116B enzymes	7](#_Toc192689922)

[Spectroscopic characterisation of CYP116B305	10](#_Toc192689923)

[CYP116B305 SDS-PAGE analysis	10](#_Toc192689924)

[FMN Quantification	11](#_Toc192689925)

[Substrate binding	13](#_Toc192689926)

[Kinetic analysis	14](#_Toc192689928)

[*In vitro* turnover of 2-HPA by CYP116B305	15](#_Toc192689930)

[*In vitro* turnover of HPPA by CYP116B305	16](#_Toc192689931)

[*In vitro* turnover of 4-methoxyacetophenone by CYP116B305	16](#_Toc192689933)

[Spectroelectrochemistry	17](#_Toc192689935)

[References	18](#_Toc192689936)

# CYP116B305 protein sequence

MAEITSIGACPFAQGEAEAALARPFDPFGRDYMEDPAGFVRRAAGERPIFFAPALGYWVVTRYDTIKAIFRDPWTFSPANVLEPVVPHSQEALAVLRRYGYAMARTLVNEDEPQHMQRRRVLMAPFTPEHLAAHEPFVRRLVREAVDGFVELGRVDLVERLLWPVPFTVALHFLGIDEEDRARMKRFSIAHTVNAFGRPTPEERLAIAETVGQFWQFSGEILEKMRRTPDGPGWMRYSIRQQKLYPEIVTDSYLHSMMMAIIVAAHETTAFAAANAVRTLLSHPTAWRELHEEPALISPAVEECLRLEGSIASWRRRTTREVEVEGVRLPAGANLLMVVAAANRDPARFPDPENLDIRRDNAQDHLTFGFGAHQCLGKNLGRMEIQIMLEELTRRLPHLQLAPQDFEYVPNLSFRGPQHLWVEWDPAVNPERRDPSLRMHRHPVRIGAPSARDRVRSVRVRALERLARDVVGLVLESLDGAPLPPWTPGAHIEVECGDPERARAYSLCSDPADRRSWRIAVLREPAGRGGSAWIHETVHPGTILKVRGPRNRFPFDERATGPVLFLAGGIGVTPLLPMAARARALGLDYRLVYCGRSRARMAFLEELLALHGPRLELAVSEEGSRLDLEALLGALPPTVRIWACGPVRMLDALERLLGDRSEAVLVTERFAATAARPAAAGERTFEVVCAHSGLTLAVPPERTLLEVLRAANLDVASDCEEGLCGTCEVQVLEGEVDHRDAVLSRAERARSDRMMACCSRARGSRLVLAL

# Protein Composition and Stability

The high protein sequence identity of CYP116B305 with the thermophilic CYP116 enzymes prompted analysis of the amino acid composition to identify potential attributes contributing to thermal adaptation. Studies on thermophilic proteins have identified key compositional trends, including lower Ala content and higher Ile content compared to mesophilic proteins (Chang and Loew 2000). Whilst the Ala content in CYP116B305 (12.3%) closely matches that of mesophilic P450 RhF (12.4%), the Ile content is slightly higher (3.2% *vs* 2.8%). Another major feature that has been linked to thermal stability of enzymes is the ratio of charged residues (Asp, Glu, Lys, His, Arg) to polar uncharged residues (Asn, Gln, Ser, Thr) (Das and Gerstein 2000; Rahban et al. 2022). CYP116B305 exhibited a higher ratio than that of mesophilic P450 RhF and positions it in between other thermophilic CYP116B members (Tavanti et al. 2018) with T_50_ values ranging from 57°C - 60°C (**Table S1**). However, this feature does not seem to directly correlate with thermal stability. For instance, CYP116B29 has the highest ratio among all CYP116B enzymes yet shares a similar T_50_ value with others.

**Table S1** Comparison of the ratio of charged residues (Asp, Glu, Lys, His, Arg) to polar uncharged residues (Asn, Gln, Ser, Thr), as well as T_50_ values, of mesophilic P450 RhF and thermophilic CYP116B members, including the newly characterised CYP116B305.

| **P450** | **T_50_** | **Ratio** |
| --- | --- | --- |
| P450 RhF (CYP116B2) | - | 28.3:15.0% (1.9) |
| CYP116B64 | 57.3°C | 29.0:14.4% (2.0) |
| CYP116B46 | 60.3°C | 31.3:15.5% (2.0) |
| **CYP116B305** | **57**°C | **28.8:11.8% (2.4)** |
| CYP116B29 | 59.3°C | 31.6:11.3% (2.8) |

**Table S2** Comparison of the active-site residues of CYP116B enzymes, including the newly characterised CYP116B305. Residues that differ in the thermophilic enzymes (CYP116B46 and CYP116B305) are highlighted in bold.

| **CYP116B2** | **CYP116B234** | **CYP116B5** | **OhpA** | **CYP116B46** | **CYP116B305** |
| --- | --- | --- | --- | --- | --- |
| Val 115 | Val 111 | Val 107 | Val 121 | Val 118 | Val 108 |
| Ala 271 | Ala 267 | Ala 263 | Ala 277 | Ala 274 | Ala 264 |
| Thr 275 | Thr 271 | Thr 267 | Thr 281 | Thr 278 | Thr 268 |
| Ser 317 | Ser 313 | Ser 309 | Ser 323 | **Pro 320** | Ser 310 |
| Val 318 | Val 314 | Val 310 | Val 324 | Val 321 | **Ile 311** |
| Trp 321 | Trp 317 | Trp 313 | Trp 327 | Trp 324 | Trp 314 |
| Ala88 | Ala 84 | Ala 80 | Ala 94 | **Val 91** | **Val 81** |
| Thr 113 | Thr 109 | Thr 105 | Thr 119 | Thr 116 | Thr 106 |
| Ala 267 | Ala 263 | Ala 259 | Ala 273 | Ala 270 | Ala 260 |
| Val 270 | Val 266 | Val 262 | Val 276 | Val 273 | Val 263 |
| Phe 422 | Phe 418 | Phe 413 | Phe 427 | Phe 424 | Phe 414 |
| Leu 89 | Leu 85 | Leu 81 | Leu 95 | Leu 92 | Leu 82 |
| Trp 203 | Trp 199 | Trp 195 | Trp 209 | **Phe 206** | **Phe 196** |
| Glu 274 | Glu 270 | Glu 266 | Glu 280 | Glu 277 | Glu 267 |
| His 198 | His 194 | His 190 | His 204 | His 201 | His 191 |
| Thr 199 | Thr 195 | Thr 191 | Thr 205 | Thr 202 | Thr 192 |
| Asn 201 | Asn 197 | Asn 193 | Asn 207 | Asn 204 | Asn 194 |
| Thr 202 | Thr 198 | Thr 194 | Thr 208 | **Ala 205** | **Ala 195** |

| **Substrate** | **Hydrophilic (hydrogen bonding)** | **Hydrophobic** |
| --- | --- | --- |
| 2-HPA | Val108, Ile261 | Val108, Ala264, Ala260, Trp314 |
| HPPA | Leu107, Val108 | Val108, Ala264, Trp314, Phe414 |

**Table S3** Identification of key binding interactions between CYP116B305 and the substrates, 2-HPA and HPPA.

# Amino acid sequence alignment of CYP116B enzymes

**Fig. S1** The amino acid sequence alignment of characterised CYP116B subfamily enzymes performed using Clustal Omega web server and visualized by ESpript 3.0 software. The secondary structure elements presented on top.

# Spectroscopic characterisation of CYP116B305

**Fig. S2** UV-visible spectra (250 nm - 700 nm) of purified CYP116B305. The observed Reinheitszahl (RZ, A_418_/A_280_) is 0.82. While an RZ >1 is typically indicative of pure bacterial P450 proteins, fused self-sufficient P450 enzymes often exhibit lower RZ values due to their larger size, which increases absorbance at 280 nm.

# CYP116B305 SDS-PAGE analysis

**Fig. S3** SDS-PAGE (NuPAGE 4 – 12%) analysis of CYP116B305 protein purified using Ni-NTA affinity chromatography. Masses of the protein ladder (ThermoFisher, Novex Sharp Pre-Stained Protein Standard) are indicated.

# FMN Quantification

**Fig. S4** UV-visible spectra (250 – 600 nm) of FMN released after heating CYP116B305 enzyme (~ 20 µM) at > 95℃ for 5 min (n=2). Two characteristic peaks at ∼375 nm and ∼445 nm, indicating a clear presence of an oxidised form of FMN cofactor. Absorbance at 445 nm = 0.083 (ε_445 nm_ = 12500 M⁻^1^ cm⁻^1^) corresponding to ~ 6.6 µM of FMN.

**Fig. S5** CO-complex formation of purified CYP116B305 (1 μM) following reduction with sodium dithionite (2 mM; pink) and NADPH (2 mM; black) in the presence of substrate (2-HPA; 1 mM).

**Fig. S6** UV-visible spectra (300 – 700 nm) of purified CYP116B305 (1 µM) in its oxidised state (black), substrate-free reduced state (green) and substrate-bound (2-HPA) reduced state (blue).

# Substrate binding

**Fig. S7** UV-visible spectra of purified CYP116B305 (black) and in the presence of various substrates (pink): **(a)** phenylacetic acid, **(b)** 4-methoxyacetophenone, **(c)** salicylic acid, **(d)** 4-hydroxyphenylacetic acid, and **(e)** trans-cinnamic acid. Insets show binding curves, where absorbance differences between the peak and trough in the spectral shifts were plotted against increasing substrate concentrations. The data were fitted to the Michaelis-Menten equation (Eq. 2) using GraphPad Prism to determine K_D_ values.

# Kinetic analysis

**Fig. S8** Kinetic parameters of CYP116B305 determined using substrate-dependent **(a)** NADPH and **(b)** NADH oxidation in the presence of the substrate 2-HPA. Error bars represent standard deviation (n = 3).

**Fig. S9** Kinetic parameters of CYP116B305 investigated using cytochrome c as an artificial electron acceptor, and NADPH as an electron donor. The apparent K_M_ and k_cat_ values for NADPH were observed to be 1.2 ± 0.1 µM and 4.1 ± 0.1 min^-1^, respectively.

# *In vitro* turnover of 2-HPA by CYP116B305

**Fig. S10** **(a)** GC Chromatogram of *in vitro* turnover of 2-HPA by CYP116B305 in the presence of NADH. The internal standard (IS; HPPA) is as indicated. Products were extracted into ethyl acetate and derivatised using BSTFA-TMS before GC-MS analysis. TMS derivatised homogentisic acid standard with retention time 15.2 min is shown in orange. **(b)** Mass spectrum of BSTFA-TMS derivatised turnover product (homogentisic acid) with a retention time of 15.2 min, and **(c)** Mass spectrum of derivatised homogentisic acid standard with a retention time of 15.2 min.

# *In vitro* turnover of HPPA by CYP116B305

**Fig. S11** **(a)** GC Chromatogram of in vitro turnover of HPPA by CYP116B305 in the presence of NADH at 45℃. The internal standard (IS; 2-HPA) is as indicated. Products were extracted into ethyl acetate and derivatised using BSTFA-TMS before GC-MS analysis, and **(b)** Mass spectrum of BSTFA-TMS derivatised turnover product (3-(2,5-dihydroxyphenyl) propionic acid) with a retention time of 15.9 min.

# *In vitro* turnover of 4-methoxyacetophenone by CYP116B305

**Fig. S12**: Mass spectrum of BSTFA-TMS derivatised **(a)** turnover product (4-hydroxyacetophenone) with a retention time of 12.4 min, and **(b)** 4-hydroxyacetophenone in the NIST library.

# Spectroelectrochemistry

**Scheme S1** Inorganic redox mediator complexes used in this work and their redox potentials (mV vs. NHE at pH 7) (Bernhardt et al. 2006).

**Fig. S13** UV-visible spectra (300 - 800 nm) of spectroelectrochemical titration of substrate-free CYP116B305 (66 µM) at 20°C showing both oxidised (blue) and reduced (red) states. Inset selected single wavelength absorption values due to applied potential.

# References

Bernhardt PV, Chen K-I, Sharpe PC (2006) Transition metal complexes as mediator-titrants in protein redox potentiometry. JBIC J Biol Inorg Chem 11:930–936. https://doi.org/10.1007/s00775-006-0148-z

Chang Y-T, Loew G (2000) Homology Modeling, Molecular Dynamics Simulations, and Analysis of CYP119, a P450 Enzyme from Extreme Acidothermophilic Archaeon *Sulfolobus solfataricus*. Biochemistry 39:2484–2498. https://doi.org/10.1021/bi991966u

Das R, Gerstein M (2000) The stability of thermophilic proteins: a study based on comprehensive genome comparison. Funct Integr Genomics 1:76–88. https://doi.org/10.1007/s101420000003

Rahban M, Zolghadri S, Salehi N, Ahmad F, Haertlé T, Rezaei-Ghaleh N, Sawyer L, Saboury AA (2022) Thermal stability enhancement: Fundamental concepts of protein engineering strategies to manipulate the flexible structure. Int J Biol Macromol 214:642–654. https://doi.org/10.1016/j.ijbiomac.2022.06.154

Tavanti M, Porter JL, Sabatini S, Turner NJ, Flitsch SL (2018) Panel of New Thermostable CYP116B Self-Sufficient Cytochrome P450 Monooxygenases that Catalyze C−H Activation with a Diverse Substrate Scope. ChemCatChem 10:1042–1051. https://doi.org/10.1002/cctc.201701510
